# Supplementary material for: Discovery and mechanism-guided engineering of BHET hydrolases for improved PET recycling and upcycling
Source: Nat Commun. 2023 Jul 13;14:4169. doi: 10.1038/s41467-023-39929-w (PMC10344914; doi:10.1038/s41467-023-39929-w)
Supplement: Supplementary file 1 — Supplementary Information [file 41467_2023_39929_MOESM1_ESM.pdf]

Supplementary information

Discovery and mechanism-guided engineering of BHET hydrolases for improved PET recycling and upcycling

Anni Li[1,#], Yijie Sheng[1,#], Haiyang Cui[2,3,#], Minghui Wang[1], Luxuan Wu[1], Yibo Song[1], Rongrong Yang[1], Xiujuan Li[1], He Huang[1]**

[1]School of Food Science and Pharmaceutical Engineering, Nanjing Normal University, Nanjing 210009, People's Republic of China

[2]RWTH Aachen University, Templergraben 55, Aachen 52062, Germany

[3]Current Address: University of Illinois at Urbana-Champaign, Carl R. Woese Institute for Genomic Biology, 1206 West Gregory Drive, Urbana, IL 61801, USA

*Corresponding authors:

Xiujuan Li, Email: lixiujuan@njnu.edu.cn;

He Huang, Email: huangh@njnu.edu.cn

[#]These authors contributed equally to this work.

Content

Supplementary Note 1 (Phase I. Microorganism determination through screening for PET-degrading microorganisms using robust grading strategy) 6
Supplementary Note 2 (Phase II. Enzyme identification through SSN prediction of PET hydrolase with BHETase activity) 6
Supplementary Note 3 (Phase III. Comprehensive enzyme characterization with *in vitro* experiments and *in silico* molecular simulation) 7
Supplementary Note 4 (Genome sequencing of *Chryseobacterium sp.* PET-29)**....** 8
Supplementary Note 5 (Gene construction and protein expression) 8
Supplementary Note 6 (The optimal variants obtained through mechanism-guided barrier engineering) **...** 8
Supplementary Note 7 (Structure and solvation phenomenon analysis) 9
Supplementary Note 8 (Analysis of the molecular docking of ΔBsEst and ΔChryBHETase to model substrate 2PET) 10
Supplementary Note 9 (The performance of a two-enzyme degradation system in mild conditions) 10
Supplementary Note 10 (The open-loop PET upcycling to produce the high-value derivative from post-consumer plastic products) 11

Supplementary 16S rDNA gene sequence**...** ***13***
16S rDNA Sequence 1. *Bacillus subtilis* PET-86 13
16S rDNA Sequence 2. *Chryseobacterium sp.* PET-29 14

Supplementary enzyme sequences cloned in this study... ***15***
Sequence 1. Nucleotide sequence of BsEst constructed in *E. coli* 15
Sequence 2. Expressed amino acid sequence BsEst by *E. coli*.............................. 15
Sequence 3. Nucleotide sequence of ChryBHETase constructed in *E. coli* 16
Sequence 4. Expressed amino acid sequence ChryBHETase by *E. coli*................. 16
Sequence 5. The reported sequences with PET degradability used in this study 17

Supplementary References **...** ***72***

Supplementary Fig. 1 Screening and purification of the strains that capable of growing on PET powder plate.. 23
Supplementary Fig. 2 The performance of ten microbes with MHET concentration over 55 μg/L during degrading PET film.. 24
Supplementary Fig. 3 Phylogenetic tree based on 16S rRNA gene sequences. 25
Supplementary Fig. 4 Genome analysis of *Chryseobacterium sp.* PET-29. 26
Supplementary Fig. 5 Sequence identity results of ChryBHETase with other reported PET enzymes. 27
Supplementary Fig. 6 Heterologous expression of BsEst, ΔBsEst, ChryBHETase, and ΔChryBHETase mediated by PelB Signal peptides and Protein purification............. 28
Supplementary Fig. 7 The substrate specificity of BsEst and ChryBHETase. 29

Supplementary Fig. 8 The activities of BSLA upon BHET......................................30
Supplementary Fig. 9 The comparison of BsEst and Bs2Est...................................31
Supplementary Fig. 10 The sequence alignment of TfCa, BsEst and ChryBHETase. 32
Supplementary Fig. 11 Root mean square deviation (RMSD) of BsEst and ΔBsEst backbone with respect to the initial structure as a function of time with/without BHET. 33
Supplementary Fig. 12 Root mean square deviation (RMSD) of ChryBHETase and ΔChryBHETase backbone with respect to the initial structure as a function of time with/without BHET..34
Supplementary Fig. 13 Time-average RMSD of ΔBsEst and ΔChryBHETase determined from the last 40 ns with/without BHET...35
Supplementary Fig. 14 The radius of gyration (R_g) of (a) BsEst and ChryBHETase and (b) ΔBsEst and ΔChryBHETase determined from the last 40 ns with/without BHET. 36
Supplementary Fig. 15 The number of internal hydrogen bonds of (a) BsEst and ChryBHETase and (b) ΔBsEst and ΔChryBHETase with > 95 % occupancy determined from the last 40 ns with/without BHET..37
Supplementary Fig. 16 The time-averaged total SASA, hydrophobic SASA, and hydrophilic SASA of BsEst and ΔBsEst determined from the last 40 ns with/without BHET. 38
Supplementary Fig. 17 The time-averaged total SASA, hydrophobic SASA, and hydrophilic SASA of ChryBHETase and ΔChryBHETase determined from the last 40 ns with/without BHET. 39
Supplementary Fig. 18 The number of water molecules at the substrate binding site and truncated region of (a) BsEst and (b) ChryBHETase with/without BHET. 40
Supplementary Fig. 19 The distance of BsEst and ΔBsEst active site with/without BHET. 41
Supplementary Fig. 20 The distance of ChryBHETase and ΔChryBHETase active site with/without BHET...42
Supplementary Fig. 21 The B-factor of the optimal ΔBHETase and BHETase........43
Supplementary Fig. 22 Binding mode analysis of BsEst and its truncated variants with BHET. 44
Supplementary Fig. 23 Binding mode analysis of ChryBHETase and its truncated variants with BHET...45
Supplementary Fig. 24 Thermostability of ΔBsEst, ΔChryBHETase and their wide type from 30 °C to 70 °C. 46
Supplementary Fig. 25 RMSF of BsEst truncated region (residue L410 to K418) and ChryBHETase truncated region (residue V66 to K76). Data plotted from the average of three independent MD runs..47
Supplementary Fig. 26 Changes in the position of overall structure of ΔBsEst and ΔChryBHETase between the active sites and BHET from 0-100 ns. 48

Supplementary Fig. 27 Binding mode analysis of ΔBsEst, ΔChryBHETase and their wide type with 2PET. 49
Supplementary Fig. 28 The binding energy results of different ligands (2PET, BHET, MHET, and TPA) with ΔBsEst, BsEst, ΔChryBHETase and ChryBHETase in molecular docking. 50
Supplementary Fig. 29 RMSF of ΔBsEst and ΔChryBHETase residues determined from the last 40 ns of MD with/without BHET. Data plotted from the average of three independent MD runs. 51
Supplementary Fig. 30 Electrostatic potential energy distribution of (a) ΔBsEst and (b) ΔChryBHETase. 52
Supplementary Fig. 31 High Performance Liquid Chromatography (HPLC) data of homogeneous TPA. 53
Supplementary Fig. 32 The reaction curve of ΔBHETases and other PET hydrolases. (a) Depletion curve of BHET as a substrate. 54
Supplementary Fig. 33 Two-enzyme degradation system consisting of PETase and ΔBHETase. 55
Supplementary Fig. 34 Depolymerization of commercial post-consumed PET products into BHET and upgrading of the pure monomers into a high-value derivative *p*-Phthaloyl chloride. 56
Supplementary Fig. 35 Liquid chromatography-mass spectrometry (LC-MS) data of BHET, TPA, and DMT. (a) LC-MS data of BHET by chemical glycolysis; (b) LC-MS data of TPA after enzymatic catalysis of truncated variants; (c) LC-MS data of DMT synthesized from TPA. (d) LC-MS data of *p*-phthaloyl chloride synthesized from TPA. 58
Supplementary Fig. 36 DSC trace of virgin PET regenerated from the degraded solutions. 59

Supplementary Table 1. Summary and classification of strains with over 55 μg/L MHET. 60
Supplementary Table 2. The genome used to perform SSNs analysis in this study..61
Supplementary Table 3. Genome characteristics of strain *Chryseobacterium sp.* PET-29 62
Supplementary Table 4. The enzyme in *Chryseobacterium sp.* PET-29 that are speculated to be involved in PET hydrolysis. 63
Supplementary Table 5. The enzymes in *Bacillus subtilis* PET-86 that are speculated to be involved in PET hydrolysis. 64
Supplementary Table 6. Common structural features of PET hydrolases. 65
Supplementary Table 7. Twenty-nine reported PET hydrolases are used to sequence similarity networks 66
Supplementary Table 8. Information of enzymes cloned in this study 67
Supplementary Table 9. Half-life of BHETases and ΔBHETases at different temperatures[a] 68

Supplementary Table 10. Summary of calculated observables during MD simulation. 69
Supplementary Table 11. Twenty-one commercial post-consumed plastic products collected in this study [a]. 70
Supplementary Table 12. The primers of target gene cloning and pET-22b(+) vector. 71

Supplementary Note 1 (Phase I. Microorganism determination through screening for PET-degrading microorganisms using robust grading strategy)

We collected 50 samples from the refuse landfill, including PET bottles, PET boxes, PET film, PET sheets, and other debris with the surrounding soil. The easily degradable diethyl phthalate (DET) was chosen as the sole carbon source in the early stage of the easy-to-hard stepwise screening system, followed by PET powder for strain separation and isolation (Supplementary Fig. 1). Approximately 70 microbes capable of growing with DET were validated (Fig. 1c). Then, the accumulation of product MHET, an intermediate degrading BHET to TPA, was selected as a screening marker to evaluate the degradation effect. Afterward, we successfully isolated ten bacteria capable of degrading and assimilating PET with MHET product over 55 μg/L (Supplementary Fig. 2). 16S rDNA gene sequencing classified them into *Bacillus*, *Chryseobacterium*, *Pseudomonas*, *Klebsiella,* and *Enterobacter* (Supplementary Table 1). Regarding the degradation performances and phylogenetic tree analysis (Supplementary Fig. 2-3), Strain No.70 and Strain No.29 had efficient degradability in 48h (162.58 μg/L, Supplementary Fig. 2) and the highest MHET yield in 8 days (197.08 μg/L, Supplementary Fig. 2), thereby determined for further study. Strain No.70 was identified as *Bacillus subtilis* PET-86 (GenBank ID: OP564167), agreeing well with a previous study that *Bacillus subtilis* TB8 leads to the weight loss of PET film[1]. Strain No.29 represents a novel species of the genus *Chryseobacterium*, for which we propose the name *Chryseobacterium sp.* PET-29 (GenBank ID: OP564169). Both *Bacillus subtilis* PET-86 and *Chryseobacterium sp.* PET-29 can grow on olive oil plates by producing prominent hydrolysis circles (Fig. 1d-e), implying that there are secreted hydrolases that can break ester bonds in olive oil and/or PET.

Supplementary Note 2 (Phase II. Enzyme identification through SSN prediction of PET hydrolase with BHETase activity)

Through screening the NCBI database and genome sequence, we obtained the whole genome of Bacillus subtilis PET-86 (Genbank: CP053102) and Chryseobacterium sp. PET-29 (Genbank: CP107053, Fig. 2a, Supplementary Fig. 4 and Supplementary Table 3). Regarding PET hydrolases mainly belong to carboxylic ester hydrolases with EC 3.1.1.X [2], we identified 48 and 37 potential PET hydrolases with α/β-structural domains from the identified open reading frame (ORF) of Bacillus subtilis PET-86 and Chryseobacterium sp. PET-29, respectively (Table S4 and S5). Experimental characterizations of all potential PET hydrolases are unfeasible. Therefore, we employed sequence similarity networks (SSNs) to capture uncharacterized enzymes by clustering them with known sequence-function space (29 reported PET hydrolases in Table S7). SSNs (EFI-EST, https://efi.igb.illinois.edu/efi-est/) are visually powerful tools for analyzing sequence relationships in "favorite" protein families [3]. As shown in

Fig. 2b, only two potential enzymes, QJR48005.1 and assembly_03435, were clustering together with BS2Est and Chath_Est1 with PET degradability[4–6]. And QJR48005.1 showed 95.7% protein sequence identity with BS2Est, suggesting extra substitutions exist in QJR48005.1 (named as BsEst, Supplementary Fig. 9). However, there was only max. 25.44% identity was found for assembly_03435 (named ChryBHETase) compared to 28 PET hydrolases (Supplementary Fig. 10).

Supplementary Note 3 (Phase III. Comprehensive enzyme characterization with *in vitro* experiments and *in silico* molecular simulation)

In terms of *in vitro* characterization, QJR48005.1 and assembly_03435 were successfully constructed and expressed in *E.coli* BL21(DE3). The crude enzymes were first examined for PET film. However, no detectable monomer was observed within 5 days, indicating both enzymes unable to degrade PET directly. These results confirmed our SSN analysis that no PETase-relevant enzyme can be founded in both PET-biodegrading strains (Fig. 2b). Instead, QJR48005.1 and assembly_03435 enable to catalyze the hydrolyzation of BHET to MHET and further to TPA (Fig. 2d, e). Different from QJR48005.1, the catalytic preference of assembly_03435 for BHET was over *p*NP-aliphatic esters (*p*NPB (C_4), *p*NPC (C_8), and *p*NPP (C_{16}), Supplementary Fig. 7a). And BHET can be converted entirely to MHET and TPA within 12 h (Fig. 2d-e). Furthermore, to verify the higher specificity of QJR48005.1 and assembly_03435 for BHET, lipase A from *Bacillus subtilis* (BSLA) were used to catalyze BHET hydrolysis. BSLA has a wide/shallow substrate binding cleft (SBC) around the active site without a "lid" domain. Theoretically, it may possess a solid hydrolytic activity for PET or BHET. However, degradation data on BHET substrates was beyond our expectations. In Supplementary Fig. 8, the BHET conversion of QJR48005.1 (100%) and assembly_03435 (46.92%) was almost 5-fold and 2.2-fold higher than that of BSLA (21.8%). And no TPA can be detected in BSLA degradation, which differs from QJR48005.1 and assembly_03435. Thus, the assembly_03435 protein prefers BHET to aliphatic esters, compared with PETase and MHETase [7,8], leading to its designation as the BHET hydrolase (termed BHETase). Therefore, we assign the name ChryBHETase for assembly_03435. Notably, QJR48005.1, named BsEst, had higher BHET conversion activities than its homology Bs2Est. In detail, the BHET conversion of BsEst is near 100% in 9 h, yet Bs2Est merely achieves 76.9% (Supplementary Fig. 9a), suggesting the 21 substitutions in BsEst are favorable to BHETase activity. Additionally, the substrate specificity of BHETases for other phenyl esters, glycerides, and tertiary alcohols was also investigated in Supplementary Fig. 7b. Results indicated that BsEst showed significant hydrolysis of almost all substrates tested in this study, while ChryBHETase showed significant hydrolysis of 2-methylbutyl acetate, glyceryl tributyrate and olive oil substrates.

Supplementary Note 4 (Genome sequencing of *Chryseobacterium sp.* PET-29)

The homology genomes of *Bacillus subtilis* PET-86 were obtained through NCBI database (Table S2). In particular, due to the lack of a homologous genome of *Chryseobacterium sp.* PET-29, the superior performances of *Chryseobacterium sp.* PET-29 compelled us to examine its underlying carboxylesterases gene through genome sequencing. As shown in Fig. 2a, Supplementary Fig. 4, and Supplementary Table 3, the whole genome of *Chryseobacterium sp.* PET-29 had a single circular chromosome containing 4,125,210 bases with a G + C content of 37.69% and annotated 3645 protein-coding sequences, 15 rRNAs, 63 tRNAs, and 26 misc RNA. In total, 2960, 1246, 808, 1664, 1608, 1868, and 2972 genes were successfully annotated by the NR, COG, KEGG, Uniprot, GO, Tigrfam, and Pfam databases, respectively (Supplementary Fig. 3).

Supplementary Note 5 (Gene construction and protein expression)

Owing to the presence of signal peptide PelB in pET-22b(+), there is good soluble protein expression in the cytoplasm. After Ni-affinity purification, the yields of BsEst and ChryBHETase in the cytosol were determined to be 1860 and 220mg/L, respectively (Supplementary Fig. 6 and Supplementary Table 10).

Supplementary Note 6 (The optimal variants obtained through mechanism-guided barrier engineering)

Based on the above structural and solvation observables, we empirically generated five truncated variants of each enzyme by deletingV60-R77 (BsEst-Δ1), G105-A112 (BsEst-Δ2), K267-F275 (BsEst-Δ3), L401-K418 (BsEst-Δ4), L410-K418 (BsEst-Δ5) of BsEst and P63-D78 (ChryBHETase-Δ1), V66-K76 (ChryBHETase-Δ2), G267-F274 (ChryBHETase-Δ3), Q319-P327 (ChryBHETase-Δ4), S368-G376 (ChryBHETase-Δ5)of ChryBHETase, respectively (Supplementary Fig. 22-23). In this study, the 3D structures of each variant were predicted by AlphaFold2. Subsequently, the binding energy, number of hydrogen bonds, serine affinity attack distance, and cavity volume of each variant were calculated by molecular docking (Supplementary Fig. 11-12). However, introducing truncation to enzymes may have an unclear impact on enzyme activity and stability, so it is necessary to call for knowledge-guided rules (normalized scoring) to improve library quality and reduce screening efforts. According to the mechanism of the catalytic triad, the nucleophilic attack distance of the Ser from the carbonyl carbon atom on the substrate determines the effective catalytic activity and is therefore assigned a maximum weight of 40%; the generation of hydrogen bonding forces between the substrate and the surrounding amino group is essential to maintain

the stability of the substrate binding and is therefore assigned a weight of 30%; the cavity volume formed by the substrate with the surrounding residues may facilitate the swinging entry of the PET chain, earning the weight of 20%. Based on normalized scoring (Eq. 5), the optimal variants of BsEst-Δ5 and ChryBHETase-Δ2 were 0.57 and 0.67, respectively, which were all higher than their wide type of 0.40 and 0.24 (Fig. 3c). The optimal variants obtained by normalized scoring are named ΔBsEst and ΔChryBHETase if not otherwise specified in this study.

Supplementary Note 7 (Structure and solvation phenomenon analysis)

BsEst and ChryBHETase have a hydrophobic SBC whose shape, size, depth, and physicochemical properties of the hydrophobic interaction zone ensure critical interactions with substrates [2]. Some studies also showed that extra "lid" domains usually negatively affect the activity of enzymes such as lipase and cutinase [2]. Previous reports have indicated that protein engineering, including enlarging the opening size of SBC [9,10], opening lids [11], and site-directed mutagenesis in lid domain [12] can increase enzyme activity and thermostability.

As shown in Supplementary Fig. 12-14, RMSD of BsEst, ChryBHETase, ΔBsEst and ΔChryBHETase in without BHET system and with BHET system for 100 ns indicated that the RMSD values remain stable in the last 40 ns of the simulation and no more large overall fluctuations occurred. The time-average RMSD of ΔBsEst slightly decreases BHET with water system while ΔChryBHETase increases slightly and changes within 0.4 Å. The radius of gyration (R_g) and the time-averaged total SASA, hydrophobic SASA, and hydrophilic SASA of BsEst, ChryBHETase, ΔBsEst and ΔChryBHETase showed essentially no change, as shown in Supplementary Fig. 16-17. Towards the number of internal hydrogen bonds in Supplementary Fig. 15, BHET with water system all showed a slight increase, indicating that adding BHET increased the number of hydrogen bonding interactions in BHETase. As shown in Supplementary Fig. 29, the RMSF of ΔBsEst and ΔChryBHETase residues were overall lower in the BHET-water system than the water system, demonstrating that the protein structure is more stable in the BHET-water system. As shown in Supplementary Fig. 18, the number of water molecules in both SBC of BsEst and ChryBHETase decreased in the BHET-water system as BHET replaced water molecules. As shown in Supplementary Fig. 25-26, the distance between the active sites of the truncated variant ΔBsEst decreased, while ΔChryBHETase showed the opposite trend, which may be due to the interaction between the substrate and the active site.

Supplementary Note 8 (Analysis of the molecular docking of ΔBsEst and ΔChryBHETase to model substrate 2PET)

To further confirm the conformational changes of different substrates in SBCs after truncation and interaction with the surrounding residues, different ligands (2PET, BHET, MHET, and TPA) were docked into BsEst, ΔBsEst, ΔChryBHETase, and ChryBHETase using Autodock, respectively, in which optimal models are selected for visualization and calculated corresponding binding energies (Supplementary Fig. 27-28). The docking results showed that binding energy for substrates (BHET, MHET, and TPA) changes remarkably after truncation. The binding energy of the PET model substrate (2PET) differed significantly. Supplementary Fig. 27 showed the binding features of 2PET to the active sites, and hydrogen bond interactions were formed between 2PET and surrounding residues. Compared with BsEst, the binding energy of ΔBsEst for 2PET (-6.7 to -7.1 kcal/mol) was decreased, and binding energy for MHET (-6.0 to -5.6 kcal/mol) and TPA (-5.7 to -5.3 kcal/mol) was enhanced (Supplementary Fig. 28). The 2PET was considerably stabilized by hydrogen bond interactions (6 hydrogen bonds) in ΔBsEst, whereas four hydrogen bond interactions were formed between 2PET and BsEst. The results indicated that the improved catalytic efficiency collectively resulted from enhancing the ability of substrate binding and product release. Also, the truncated ΔBsEst may enhance affinity for PET. In contrast, ΔChryBHETase with good performance seems to be attributed to significantly improving product release (-6.8 to -5.6 kcal/mol for MHET; -6.6 to -6.1 kcal/mol for TPA), despite an increase in binding energy to 2PET (-7.2 to -6.2 kcal/mol) (Supplementary Fig. 27-28).

Supplementary Note 9 (The performance of a two-enzyme degradation system in mild conditions)

After 96 h at 60 °C, the concentrations of products increased slowly with time. We speculated that the product inhibition might be the reason for this phenomenon. Notably, at 96 h, for PET hydrolases with great thermostability, including DepoPETase, FAST-PETase, and LCC-ICCG, the total amount of product released by a two-enzyme system with ΔBHETases was up to 1.3-fold compared to the single-enzyme system; For PET hydrolases with poor thermostability, such as PETase, LCC, DuraPETase and ThermoPETase, the total amount of products released by a two enzyme system with ΔBHETases was up to 1.6-fold compared to the single-enzyme system (Fig. 5b-h). This synergy effect was more significant in the case of degrading PET film. The findings in Fig. 5j provide visual and microscopic evidence of the enhanced PET degradation achieved by implementing the two-enzyme system, further validating its efficacy and potential for practical applications.

In previous studies [13–17], researchers have commonly opted for high temperatures to exploit the ability of heat to reduce the crystallinity of PET. This decrease in

crystallinity promotes plastic decomposition, rendering it more amenable to degradation processes. However, operating at elevated temperatures often incurs additional energy costs. In our endeavor to develop a more environmentally friendly process, we investigated the efficiency of PET degradation using a two-enzyme system at room temperature (30 °C). As shown in Supplementary Fig. 33a, involving BHETase and ΔBHETase in PET degradation enabled to offset of the poor ability of PETase in converting BHET to TPA. Notably, PETase+ΔBsEst can achieve a 100% yield of TPA in 4 h, but PETase+BsEst with 94.8%. Such synergy effect was more significant in the case of degrading PET film (Supplementary Fig. 33b). TPA is obtained through the action of (i) PETase alone, (ii) PETase+BsEst, (iii) PETase+ΔBsEst, (iv) PETase+ChryBHETase, and (v) PETase+ΔChryBHETase. Notably, PETase+ΔBHETase had up to 7-fold improvement in TPA yield (e.g., ΔBsEst: 663.1 μM) compared to single PETase (95 μM). In contrast, with PETase, the amounts of BHET, MHET, and TPA were measured reliably and satisfactorily determined to be 29.3 μM, 463.3 μM, and 95 μM within 24 h, respectively. As depicted in Supplementary Fig. 33b, the two-enzyme approach successfully reduced the concentration of MHET to undetectable levels. Furthermore, the highest TPA concentrations were observed with PETase+ΔBsEst, reaching 663.1 μM, compared to PETase+BsEst (617.6 μM of TPA). Similarly, PETase+ChryBHETase and PETase+ΔChryBHETase exhibited enhanced TPA production, with measured concentrations of 561.3 μM and 589.6 μM, respectively, surpassing the anticipated levels. These findings demonstrate the effectiveness of the two-enzyme system in achieving higher TPA yields compared to single-enzyme systems and underline the potential of ΔBHETases in enhancing PET degradation and TPA production. This founding was also proved by analyzing the surface of the PET film shown by visible photography, scanning electron microscopy (SEM), and the reduced water contact angle (Supplementary Fig. 33c). Although the short detection time (24 h) slightly hid the outstanding properties of ΔBHETases (e.g., 5-7.3% improved conversion compared to BHETase), PETase+ΔBHETases enabled to degrade PET film strongly, resulting in much rougher surfaces (Supplementary Fig. 33c, middle panel). Overall, the simple two-enzyme degradation system, especially with ΔBHETase, can efficiently produce pure TPA.

Supplementary Note 10 (The open-loop PET upcycling to produce the high-value derivative from post-consumer plastic products)

Subsequently, the pure TPA was upgraded into an exemplary chemical product, *p*-phthaloyl chloride, in a simple one-step method (Benzyltriethylammonium chloride (TEBAC) under 85 °C). *p*-Phthaloyl chloride is a valuable monomer for particular fiber synthesis as well as a reinforcing agent for aramid and nylon[18]. Assuming that no losses occurred during the processing of samples between ΔBHETase catalysis and two chemical processes (PET glycolysis and halogenation), the *p*-Phthaloyl chloride yields from the original plastics were on average 70.20% for 21 commercial plastic products

(Fig. 7d). Collectively, based on the engineered ΔBHETase, we demonstrated a process concept for pairing chemical and enzymatic catalysis to convert plastic waste into valuable products. Such technologies would support the creation of a circular plastics economy [19].

Supplementary 16S rDNA gene sequence

16S rDNA Sequence 1. *Bacillus subtilis* PET-86

TGGTGTGACGGGCGGTGTGTACAAGGCCCGGGAACGTATTCACCGCGGC
ATGCTGATCCGCGATTACTAGCGATTCCAGCTTCACGCAGTCGAGTTGCA
GACTGCGATCCGAACTGAGAACAGATTTGTGGGATTGGCTTAACCTCGCG
GTTTCGCTGCCCTTTGTTCTGTCCATTGTAGCACGTGTGTAGCCCAGGTCA
TAAGGGGCATGATGATTTGACGTCATCCCCACCTTCCTCCGGTTTGTCACC
GGCAGTCACCTTAGAGTGCCCAACTGAATGCTGGCAACTAAGATCAAGGG
TTGCGCTCGTTGCGGGACTTAACCCAACATCTCACGACACGAGCTGACGA
CAACCATGCACCACCTGTCACTCTGCCCCCGAAGGGGACGTCCTATCTCTA
GGATTGTCAGAGGATGTCAAGACCTGGTAAGGTTCTTCGCGTTGCTTCGA
ATTAAACCACATGCTCCACCGCTTGTGCGGGCCCCCGTCAATTCCTTTGAG
TTTCAGTCTTGCGACCGTACTCCCCAGGCGGAGTGCTTAATGCGTTAGCTG
CAGCACTAAGGGGCGGAAACCCCCTAACACTTAGCACTCATCGTTTACGG
CGTGGACTACCAGGGTATCTAATCCTGTTCGCTCCCCACGCTTTCGCTCCT
CAGCGTCAGTTACAGACCAGAGAGTCGCCTTCGCCACTGGTGTTCCTCCA
CATCTCTACGCATTTCACCGCTACACGTGGAATTCCACTCTCCTCTTCTGC
ACTCAAGTTCCCCAGTTTCCAATGACCCTCCCCGGTTGAGCCGGGGGCTTT
CACATCAGACTTAAGAAACCGCCTGCGAGCCCTTTACGCCCAATAATTCC
GGACAACGCTTGCCACCTACGTATTACCGCGGCTGCTGGCACGTAGTTAG
CCGTGGCTTTCTGGTTAGGTACCGTCAAGGTACCGCCCTATTCGAACGGTA
CTTGTTCTTCCCTAACAACAGAGCTTTACGATCCGAAAACCTTCATCACTC
ACGCGGCGTTGCTCCGTCAGACTTTCGTCCATTGCGGAAGATTCCCTACTG
CTGCCTCCCGTAGGAGTCTGGGCCGTGTCTCAGTCCCAGTGTGGCCGATCA
CCCTCTCAGGTCGGCTACGCATCGTTGCCTTGGTGAGCCATTACCTCACCA
ACTAGCTAATGCGCCGCGGGTCCATCTGTAAGTGGTAGCCGAAGCCACCT
TTTATGTTTGAACCATGCGGTTCAAACAACCATCCGGTATTAGCCCCGGTT
TCCCGGAGTTATCCCAGTCTTACAGGCAGGTTACCCACGTGTTACTCACCC
GTCCGCCGCTAACATCAGGGAGCAAG

The GenBank accession number for the 16S rDNA gene sequence of the strain PET-86 is OP564167.

16S rDNA Sequence 2. ***Chryseobacterium sp.*** **PET-29**

AGGTACCCCAGACTTCCATGGCTTGACGGGCGGTGTGTACAAGGCCCGG
GAACGTATTCACCGCGCCATGGCTGATGCGCGATTACTAGCGATTCCAGC
TTCATAGAGTCGAGTTGCAGACTCCAATCCGAACTGAGACCGGCTTTCGA
GATTTGCATCACATCGCTGTGTAGCTGCCCTCTGTACCGGCCATTGTATTA
CGTGTGTGGCCCAAGGCGTAAGGGCCGTGATGATTTGACGTCATCCCCAC
CTTCCTCTCTACTTGCGTAGGCAGTCTTACTAGAGTCCTCAACTTAATGGT
AGCAACTAGTAACAGGGGTTGCGCTCGTTGCAGGACTTAACCTAACACCT
CACGGCACGAGCTGACGACAACCATGCAGCACCTTGAAAATTGCCCGAAG
GAAGGTCTATTTCTAAACCGATCAATTCCCATTTAAGCCTTGGTAAGGTTC
CTCGCGTATCATCGAATTAAACCACATAATCCACCGCTTGTGCGGGCCCCC
GTCAATTCCTTTGAGTTTCAAACTTGCGTTCGTACTCCCCAGGTGGCTAAC
TTATCACTTTCGCTTAGTCTCTGAATCCGAAAACCCAAAAACGAGTTAGCA
TCGTTTACAGCGTGGACTACCAGGGTATCTAATCCTGTTCGCTCCCCACGC
TTTCGTCCATCAGCGTCAGTTAAGACATAGTGACCTGCCTTCGCAATTGGT
GTTCTAAGTAATATCTATGCATTTCACCGCTACACTACTTATTCCAGCCAC
TTCTACCTTACTCAAGACCTGCAGTATCAATGGCAGTTTCACAGTTAAGCT
GTGAGATTTCACCACTGACTTACAGATCCGCCTACGGACCCTTTAAACCCA
ATAAATCCGGATAACGCTTGCACCCTCCGTATTACCGCGGCTGCTGGCAC
GGAGTTAGCCGGTGCTTATTCGTACAGTACCTTCAGCTATTTACACGTAAA
TAGGTTTATCCCTGTACAAAAGAAGTTTACAACCCATAGGGCCGTCGTCCT
TCACGCGGGATGGCTGGATCAGGCGCTAACCCATTGTCCAATATTCCTCA
CTGCTGCCTCCCGTAGGAGTCTGGTCCGTGTCTCAGTACCAGTGTGGGGG
ATCACCCTCTCAGGCCCCCTAAAGATCATCGACTTGGTGAGCCGTTACCTC
ACCAACTATCTAATCTTGCGCGTGCCCATCTTTATCCACCTCAGTTTTCAA
TATAAAGTGATGCCACTCTATATATTATGGGGTATTAATCTTCCTTTCGAA
AGGCTATCCCCCTGATAAAGGCAGGTTGCACACGTGTTCCGCACCCGTAC
GCCGCTCTCAAGTCTCCGAAGA

The GenBank accession number for the 16S rDNA gene sequence of the strain PET-29 is OP564169.

Supplementary enzyme sequences cloned in this study

Sequence 1. Nucleotide sequence of BsEst constructed in *E. coli*

ATGACTCATCAAATAGTAACGACTCAATACGGCAAAATAAAAGGCACA
ACGGAAAACGGCGTACATAAGTGGAAAGGCATCCCTTATGCCAAGCCGCC
TGTCGGACAATGGCGTTTTAAAGCACCTGAGCCGCCTGAAGTGTGGGAAG
ATGTCCTTGATGCCACAGCGTACGGCCCTGTTTGCCCGCAGCCGTCTGATT
TGCTCTCACTGTCGTATGCCGAGCTGCCCCGCCAGTCCGAGGATTGCTTGT
ATGTCAATGTATTTGCGCCTGACACTCCAAGTCAAAACCTGCCTGTCATGG
TGTGGATTCACGGAGGCGCTTTTTATCTTGGAGCGGGCAGTGAGCCATTAT
ATGACGGATCAAAACTTGCGGCGCAGGGAGAGGTCATTGTCGTCACACTG
AACTATCGGCTGGGGCCGTTTGGCTTTTTGCACATGTCTTCATTTGATGAG
GCGTATTCCGATAACCTTGGGCTTTTAGACCAAGCCGCCGCACTGAAATG
GGTGCGAGAGAATATCTCAGCGTTTGGCGGTGACCCCAATAACGTAACAG
TATTTGGAGAATCCGCCGGCGGCATGAGCATTGCCGCGCTTCTCGCTATGC
CTGCGGCAAAAGGCCTGTTCCAGAAAGCGATCATGGAAAGCGGCGCTTCT
CGAACAATGACAAAAGAACAAGCGGCAAGCACTGCGGCTGCCTTTTTACA
GGTCCTTGGGATTAATGAGAGCCAGATGGACAGATTGCATACTGTAGCAG
CGGAAGATTTGCTTAAAGCGGCCGATCAGCTTCGGATCGCAGAAAAAGAA
AATATCTTTCAGCTGTTCTTCCAGCCCGCCCTTGATCCGAAAACGCTCCCT
GCTGAACCAGAAAAAGCGATCGCAGAAGGGGCTGCTTCCGGCATTCCGCT
ATTAATCGGAACAACCCGTGATGAAGGATATTTATTTTTCACCCCGGATTC
AGACGTTCATTCTCAGGAAACGCTTGATGCAGCGCTCGAGTATTTACTAG
GGAAGCCGCTGGCAGAGAAAGCTGCCGATTTGTATCCGCGTTCTTTGGAA
AGCCAAATTCATATGATGACTGATTTATTATTTTGGCGCCCTGCTGTCGCC
TATGCATCCGCACAGTCTCATTACGCCCCTGTCTGGATGTACCGGTTCGAT
TGGCACCCGGAGAAGCCGCCGTACAATAAAGCGTTTCACGCATTAGAGCT
TCCTTTTGTCTTTGGAAATCTGGACGGATTAGAACGAATGGCAAAAGCGG
AGGTTACGGATGAGGTGAAACAGCTTTCTCACTCGATACAAACAGCATGG
ATCACATTCGCCAAAACAGGAAACCCAAGCACCGAAGCTGTGAATTGGCC
AGCGTATCAGGAAGAAACAAGAGAGACGCTGATTTTAGATTCAAAGATTA
CGATCGAAAACGATCCCGAATCTGAAAAAAGGCAGAAGCTATTCCCTTCA
AAAGGAGAA

Sequence 2. Expressed amino acid sequence BsEst by *E. coli*

MTHQIVTTQYGKIKGTTENGVHKWKGIPYAKPPVGQWRFKAPEPPEVWED
VLDATAYGPVCPQPSDLLSLSYAELPRQSEDCLYVNVFAPDTPSQNLPVMVW
IHGGAFYLGAGSEPLYDGSKLAAQGEVIVVTLNYRLGPFGFLHMSSFDEAYS
DNLGLLDQAAALKWVRENISAFGGDPNNVTVFGESAGGMSIAALLAMPAAK
GLFQKAIMESGASRTMTKEQAASTAAFLQVLGINESQMDRLHTVAAEDLL
KAADQLRIAEKENIFQLFFQPALDPKTLPAEPEKAIAEGAASGIPLLIGTTRDEG

YLFFTPDSDVHSQETLDAALEYLLGKPLAEKAADLYPRSLESQIHMMTDLLF
WRPAVAYASAQSHYAPVWMYRFDWHPEKPPYNKAFHALELPFVFGNLDGL
ERMAKAEVTDEVKQLSHSIQTAWITFAKTGNPSTEAVNWPAYQEETRETLIL
DSKITIENDPESEKRQKLFPSKGEAALEHHHHHH

Sequence 3. Nucleotide sequence of ChryBHETase constructed in *E. coli*

ATGACAACACAGCAACACAAGAAAATCCATACCTTTCATACTTCTTTCG
GAACCATTCCGGCATTAAAGGGAAATGGGGTCATCAGGGCGAAAAGTATT
CGCTATGCCCGCTCTCAAAGATTTAAAAAGCCGGAACCCTTACCATATCC
GGAGCATGATGAAATTCCGGATAAAACCCCGGTATGCCCACAAAATGTAA
GTCCTCTGCTGGACAGGCTGATCCAGAAAACAGATGTGGAACAGTTTGAA
CCGGATGAATCCCCGCAGTTTCTTACCATTACACGTCCCGAACACTTTAAT
GAAAACGAAAAGCTTCCCGTTATCGTCTGGATTCATGGTGGCTCCTATGA
AATCGGATGCGGTGATCTTCCCACGTCTGATCCTTCCGTGTGGGTAAAGG
AACAGCACATTATTGTTGTTTCGGTATCGTACCGGCTCGGGCTTTTCGGTT
TTTTGGGAGGTAGTGAAGAAAGGCCGGCCAACCTGGGATTGTATGATATG
ATTGCCGCTCTCCAATGGATAAAACAATACATCCGCGACTTTGGCGGAGA
TCCTGAAAACATTACCCTGTTCGGACAGTCATCCGGAGGTGATGCCATTG
CACATCTCATGATCTCGGAAGAAACGGAAAATTTATTCCGCCATGTGATT
ATCCATAGTGCTCCGCTGGGATTCAGGATCAACAGGCAAAAGATGTCCCA
TGAGTTTTTCCTCAAGACGGATATTTTAAAAGATGAACCCGATGCGCTTAA
GATGGTAGCAGGATATCGCACTTTTTTACCTTCTTTCAGAAAATACGGATT
GAAGACCTCCATGCCATTCTGCACGCAATATGGCCATCCTCCGTTATGCCA
GGAAGAAGAAACCATGCCGAACTGGAAACAAAATGCAAAGAAGTATGAT
GTTTTAATAGGCTCCAATCAGGATGAAACGGCTTTTTACGTGAAAACCTC
GCAAACAGGCATTTATACTTATCTACCCCAAAGAATTCTGAATAAGATTG
TAAGAAAAACTACGGCGTCAATCTATGAAAAACCGGCCAAGGCGTTTGCA
GAAAATCTTGCAGCCGGTGGCGGAAATGTATACCAGTTTGTTATTCGGTCT
ACCCTAAAGAGCAATAACATCGGCGCATCGCACTGTGTAGATCTGCCTCT
GTTATTTGAAAACAAGGAAGCCTGGCAATCAGCAGAGCTGCTGAAAGATG
TTCCATGGGAATATATTCAGGAAAACGGGAAAAAGCTTCGGGCGCTCTGG
GCAGAATTTGCGAGATCCGGATCAATCTCAGAAGATTCAGAAAGGCCTGA
AATCCTTAAGCTTAAAAAAGTTAAGAAT

Sequence 4. Expressed amino acid sequence ChryBHETase by *E. coli*

MTTQQHKKIHTFHTSFGTIPALKGNGVIRAKSIRYARSQRFKKPEPLPYPEH
DEIPDKTPVCPQNVSPLLDRLIQKTDVEQFEPDESPQFLTITRPEHFNENEKLPV
IVWIHGGSYEIGCGDLPTSDPSVWVKEQHIIVVSVSYRLGLFGFLGGSEERPAN
LGLYDMIAALQWIKQYIRDFGGDPENITLFGQSSGGDAIAHLMISEETENLFR
HVIIHSAPLGFRINRQKMSHEFFLKTDILKDEPDALKMVAGYRTFLPSFRKYG
LKTSMPFCTQYGHPPLCQEEETMPNWKQNAKKYDVLIGSNQDETAFYVKTS

QTGIYTYLPQRILNKIVRKTTASIYEKPAKAFAENLAAGGGNVYQFVIRSTLKS
NNIGASHCVDLPLLFENKEAWQSAELLKDVPWEYIQENGKKLRALWAEFAR
SGSISEDSERPEILKLKKVKNAALEHHHHHH

Sequence 5. The reported sequences with PET degradability used in this study

>PETase(5XGO)

MNFPRASRLMQAAVLGGLMAVSAAATAQTNPYARGPNPTAASLEASAGPF
TVRSFTVSRPSGYGAGTVYYPTNAGGTVGAIAIVPGYTARQSSIKWWGPRLA
SHGFVVITIDTNSTLDQPSSRSSQQMAALRQVASLNGTSSSPIYGKVDTARMG
VMGWSMGGGGSLISAANNPSLKAAAPQAPWDSSTNFSSVTVPTLIFACENDSI
APVNSSALPIYDSMSRNAKQFLEINGGSHSCANSGNSNQALIGKKGVAWMKR
FMDNDTRYSTFACENPNSTRVSDFRTANCS

> SvCut(4WFI)

MRGSHHHHHHGSNPYERGPDPTEDSIEAIRGPFSVATERVSSFASGFGGGTI
YYPRETDEGTFGAVAVAPGFTASQGSMSWYGERVASQGFIVFTIDTNTRLDQ
PGQRGRQLLAALDYLVERSDRKVRERLDPNRLAVMGHSMGGGGSLEATVM
RPSLKASIPLTPWNLDKTWGQVQVPTFIIGAELDTIAPVRTHAKPFYESLPSSLP
KAYMELDGATHFAPNIPNTTIAKYVISWLKRFVDEDTRYSQFLCPNPTDRAIE
EYRSTCPYKLN

> BTA-hydrolase 1

MAVMTPRRERSSLLSRALQVTAAAATALVTAVSLAAPAHAANPYERGPNP
TDALLEASSGPFSVSEENVSRLSASGFGGGTIYYPRENNTYGAVAISPGYTGTE
ASIAWLGERIASHGFVVITIDTITTLDQPDSRAEQLNAALNHMINRASSTVRSRI
DSSRLAVMGHSMGGGGTLRLASQRPDLKAAIPLTPWHLNKNWSSVTVPTLII
GADLDTIAPVATHAKPFYNSLPSSISKAYLELDGATHFAPNIPNKIIGKYSVAW
LKRFVDNDTRYTQFLCPGPRDGLFGEVEEYRSTCPF

> BTA-hydrolase 2

MAVMTPRRERSSLLSRALRFTAAAATALVTAVSLAAPAHAANPYERGPNP
TDALLEARSGPFSVSEERASRFGADGFGGGTIYYPRENNTYGAVAISPGYTGT
QASVAWLGKRIASHGFVVITIDTNTTLDQPDSRARQLNAALDYMINDASSAV
RSRIDSSRLAVMGHSMGGGGSLRLASQRPDLKAAIPLTPWHLNKNWSSVRVP
TLIIGADLDTIAPVLTHARPFYNSLPTSISKAYLELDGATHFAPNIPNKIIGKYSV
AWLKRFVDNDTRYTQFLCPGPRDGLFGEVEEYRSTCPF

>Thh_Est

MANPYERGPNPTNSSIEALRGPFRVDEERVSRLQARGFGGGTIYYPTDNNTF
GAVAISPGYTGTQSSISWLGERLASHGFVVMTIDTNTTLDQPDSRASQLDAAL
DYMVEDSSYSVRNRIDSSRLAAMGHSMGGGGTLRLAERRPDLQAAIPLTPW
HTDKTWGSVRVPTLIIGAENDTIASVRSHSEPFYNSLPGSLDKAYLELDGASH
FAPNLSNTTIAKYSISWLKRFVDDDTRYTQFLCPGPSTGWGSDVEEYRSTCPF

>Thf42_Cut1

MANPYERGPNPTDALLEARSGPFSVSEENVSRLSASGFGGGTIYYPRENNTY
GAVAISPGYTGTEASIAWLGERIASHGFVVITIDTITTLDQPDSRAEQLNAALN
HMINRASSTVRSRIDSSRLAVMGHSMGGGGSLRLASQRPDLKAAIPLTPWHL
NKNWSSVRVPTLIIGADLDTIAPVLTHARPFYNSLPTSISKAYLELDGATHFAP
NIPNKIIGKYSVAWLKRFVDNDTRYTQFLCPGPRDGLFGEVEEYRSTCPF

> SeL (1JFR)

AANPYERGPAPTNASIEASRGPYATSQTSVSSLVASGFGGGTIYYPTSTADG
TFGAVVISPGFTAYQSSIAWLGPRLASQGFVVFTIDTNTTLDQPDSRGRQLLSA
LDYLTQRSSVRTRVDATRLGVMGHSMGGGGSLEAAKSRTSLKAAIPLTGWN
TDKTWPELRTPTLVVGADGDTVAPVATHSKPFYESLPGSLDKAYLELRGASH
FTPNTSDTTIAKYSISWLKRFIDSDTRYEQFLCPIPRPSLTIAEYRGTCPHTS

> Est119(3VIS)

HHHHHHMSVTTPRRETSLLSRALRATAAAATAVVATVALAAPAQAANPYE
RGPNPTESMLEARSGPFSVSEERASRFGADGFGGGTIYYPRENNTYGAIAISPG
YTGTQSSIAWLGERIASHGFVVIAIDTNTTLDQPDSRARQLNAALDYMLTDAS
SAVRNRIDASRLAVMGHSMGGGGTLRLASQRPDLKAAIPLTPWHLNKSWRD
ITVPTLIIGAEYDTIASVTLHSKPFYNSIPSPTDKAYLELDGASHFAPNITNKTIG
MYSVAWLKRFVDEDTRYTQFLCPGPRTGLLSDVEEYRSTCPF

>Tcur0390(lipase)

MKRTLKRALSLLPAAALAASALVAASPAQAAANPYQRGPNPTEASITAAR
GPFNTAEITVSRLSVSGFGGGKIYYPTTTSEGTFGAIAISPGFTAYWSSLEWLG
HRLASQGFVVIGIETNTTLDQPDQRGQQLLAALDYLTQRSAVRDRVDASRLA
VAGHSMGGGGSLEAAKARTSLKAAIPLAPWNLDKTWPEVRTPTLIIGGELDA
VAPVATHSIPFYNSLSNAPEKAYLELDNASHFFPNITNTQMAKYMIAWMKRFI
DDDTRYTQFLCPPPSTGLL

SDFSDARFTCPM

> PaPL(6SBN)

MPFNKKSVLALCGAGALLFSMSALANNPAPTDPGDSGGGSAYQRGPDPSV
SFLEADRGQYSVRSSRVSSLVSGFGGGTIYYPTGTTGTMGAVVVIPGFVSAES

SIDWWGPKLASYGFVVMTIDTNTGFDQPPSRARQINNALDYLVSQNSRSSSPV
RGMIDTNRLGVIGWSMGGGGTLRVASEGRIKAAIPLAPWDTTSYYASRSQAP
TLIFACESDVIAPVLQHASPFYNSLPSSIDKAFVEINGGSHYCGNGGSIYNDVLS
RFGVSWMKLHLDEDSRYKQFLCGPNHTSDSQISDYRGNCPYLE

> Ta_cut(6AID)

MRGSHHHHHHGSAPAQAANPYERGPNPTESMLEARSGPFSVSEERASRFG
ADGFGGGTIYYPRENNTYGAIAISPGYTGTQSSIAWLGERIASHGFVVIAIDTN
TTLDQPDSRARQLNAALDYMLTDASSAVRNRIDASRLAVMGHSMGGGGTLR
LASQRPDLKAAIPLTPWHLNKSWRDITVPTLIIGAEYDTIASVTLHSKPFYNSIP
SPTDKAYLELDGASHFAPNITNKTIGMYSVAWLKRFVDEDTRYTQFLCPGPR
TGLLSDVEEYRSTCPF

>Tcur1278(lipase)

MSLRKSFGLLSATAALVAGLVAAPPAQAAANPYQRGPDPTESLLRAARGPF
AVSEQSVSRLSVSGFGGGRIYYPTTTSQGTFGAIAISPGFTASWSSLAWLGPRL
ASHGFVVIGIETNTRLDQPDSRGRQLLAALDYLTQRSSVRNRVDASRLAVAG
HSMGGGGTLEAAKSRTSLKAAIPIAPWNLDKTWPEVRTPTLIIGGELDSIAPV
ATHSIPFYNSLTNAREKAYLELNNASHFFPQFSNDTMAKFMISWMKRFIDDDT
RYDQFLCPPPRAIGDISDYRDTCPHT

>Cut190(7CTS)

GPNPYERGPDPTEDSIEAIRGPFSVATERVSSFASGFGGGTIYYPRETDEGTF
GAVAVAPGFTASQGSMSWYGERVASHGFIVFTIDTNTRLDAPGQRGRQLLAA
LDYLVERSDRKVRERLDPNRLAVMGHAMGGGGSLEATVMRPSLKASIPLTP
WHLDKTWGQVQVPTFIIGAELDTIAPVSTHAKPFYESLPSSLPKAYMELCGAT
HFAPNIPNTTIAKYVISWLKRFVDEDTRYSQFLCPNPTDRAICEYRSTCPYKLN

>Thc_Cut2(5LUL)

MANPYERGPNPTDALLEASSGPFSVSEENVSRFGADGFGGGTIYYPRENNT
YGAVAISPGYTGTQASVAWLGERIASHGFVVITIDTNTTLDQPDSRARQLNAA
LDYMINDASSAVRSRIDSSRLAVMGHSMGGGGTLRLASQRPDLKAAIPLTPW
HLNKNWSSVRVPTLIIGADLDTIAPVLTHARPFYNSLPTSISKAYLELDGATHF
APNIPNKIIGKYSVAWLKRFVDNDTRYTQFLCPGPRDGLFGEVEEYRSTCPFA
LE

>Cutinase(4CG1)

ANPYERGPNPTDALLEARSGPFSVSEENVSRLSASGFGGGTIYYPRENNTYG
AVAISPGYTGTEASIAWLGERIASHGFVVITIDTITTLDQPDSRAEQLNAALNH
MINRASSTVRSRIDSSRLAVMGHSMGGGGSLRLASQRPDLKAAIPLTPWHLN
KNWSSVTVPTLIIGADLDTIAPVATHAKPFYNSLPSSISKAYLELDGATHFAPN

IPNKIIGKYSVAWLKRFVDNDTRYTQFLCPGPRDGLFGEVEEYRSTCPFYPNSS SVDKLAAALEHHHHHH

>Tha_Cut1

MANPYERGPNPTDALLEASSGPFSVSEENVSRLSASGFGGGTIYYPRENNTY GAVAISPGYTGTEASIAWLGGRIASHGFVVITIDTITTLDQPDSRAEQLNAALN HMINRASSTVRSRIDSSRLAVMGHSMGGGGTPRLASQRPDLKAAIPLTPWHL NKNRSSVTVPTLIIGADLDTIAPVATHAKPFYNSLPSSISKAYLELDGATHFAP NIPNKIIGKYSVAWLKRFVDNDTRYTQFLCPGPRDGLFGEVEEYCSTCPF

>LCC(4EB0)

SNPYQRGPNPTRSALTADGPFSVATYTVSRLSVSGFGGGVIYYPTGTSLTFG GIAMSPGYTADASSLAWLGRRLASHGFVVLVINTNSRFDYPDSRASQLSAAL NYLRTSSPSAVRARLDANRLAVAGHSMGGGGTLRIAEQNPSLKAAVPLTPW HTDKTFNTSVPVLIVGAEADTVAPVSQHAIPFYQNLPSTTPKVYVELDNASHF APNSNNAAISVYTISWMKLWVDNDTRYRQFLCNVNDPALSDFRTNNRHCQ

> Cutinase(4OYY)

QLGAIENGLESGSANACPDAILIFARGSTEPGNMGITVGPALANGLESHIRNI WIQGVGGPYDAALATNFLPRGTSQANIDEGKRLFALANQKCPNTPVVAGGY SQGAALIAAAVSELSGAVKEQVKGVALFGYTQNLQNRGGIPNYPRERTKVFC NVGDAVCTGTLIITPAHLSYTIEARGEAARFLRDRIRA

>Cutinase(1CUS)

LGRTTRDDLINGNSASCADVIFIYARGSTETGNLGTLGPSIASNLESAFGKDG VWIQGVGGAYRATLGDNALPRGTSSAAIREMLGLFQQANTKCPDATLIAGG YSQGAALAAASIEDLDSAIRDKIAGTVLFGYTKNLQNRGRIPNYPADRTKVFC NTGDLVCTGSLIVAAPHLAYGPDARGPAPEFLIEKVRAVRGSA

>Cutinase(3DCN)

AMAISDPQSSTRNELETGSSSACPKVIYIFARASTEPGNMGISAGPIVADALE RIYGANDVWVQGVGGPYLADLASNFLPDGTSSAAINEARRLFTLANTKCPNA AIVSGGYSQGTAVMAGSISGLSTTIKNQIKGVVLFGYTKNLQNLGRIPNFETSK TEVYCDIADAVCYGTLFILPAHFLYQTDAAVAAPRFLQARIG

>Cutinase(3GBS)

SPVDLQDRQLTGGDELRDGPCKPITFIFARASTEPGLLGISTGPAVCNRLKLA RSGDVACQGVGPRYTADLPSNALPEGTSQAAIAEAQGLFEQAVSKCPDTQIV AGGYSQGTAVMNGAIKRLSADVQDKIKGVVLFGYTRNAQERGQIANFPKDK VKVYCAVGDLVCLGTLIVAPPHFSYLSDTGDASDFLLSQLG

>Cutinase(1CEX)

LPTSNPAQELEARQLGRTTRDDLINGNSASCADVIFIYARGSTETGNLGTLG
PSIASNLESAFGKDGVWIQGVGGAYRATLGDNALPRGTSSAAIREMLGLFQQ
ANTKCPDATLIAGGYSQGAALAAASIEDLDSAIRDKIAGTVLFGYTKNLQNR
GRIPNYPADRTKVFCNTGDLVCTGSLIVAAPHLAYGPDARGPAPEFLIEKVRA
VRGSA

>Bs2Est(1QE3)

MTHQIVTTQYGKVKGTTENGVHKWKGIPYAKPPVGQWRFKAPEPPEVWE
DVLDATAYGSICPQPSDLLSLSYTELPRQSEDCLYVNVFAPDTPSKNLPVMV
WIHGGAFYLGAGSEPLYDGSKLAAQGEVIVVTLNYRLGPFGFLHLSSFNEAY
SDNLGLLDQAAALKWVRENISAFGGDPDNVTVFGESAGGMSIAALLAMPAA
KGLFQKAIMESGASRTMTKEQAASTSAAFLQVLGINEGQLDKLHTVSAEDLL
KAADQLRIAEKENIFQLFFQPALDPKTLPEEPEKAIAEGAASGIPLLIGTTRDEG
YLFFTPDSDVHSQETLDAALEYLLGKPLAEKVADLYPRSLESQIHMMTDLLF
WRPAVAYASAQSHYAPVWMYRFDWHPKKPPYNKAFHALELPFVFGNLDGL
ERMAKAEITDEVKQLSHTIQSAWITFAKTGNPSTEAVNWPAYHEETRETLILD
SEITIENDPESEKRQKLFPSKGE

> Chath_Est1(5A2G)

MAKQFLYDNLPVVETKAGKLRGYQWEGTYIFKGIRYARANRFQLPEEVEP
WEGVKEAASYGFVCPMLTRDHPQGELLVPHRYWPQDEDCLSLNIWSQSLDR
SAKKPVMFWIHGGAFSMGSSIEQKAYNGENMSRYGDVVVVTVNHRLNILGY
LDLSPYGERYAGSANAGQADLVAALKWVRDNIEAFGGDPDNVTIFGQSGGG
MKVSGLMQTPEADGLFHRAMIMSGVAGDVLPYSTGDSRPLIQAMLKELGLA
EQEAGRLETVPYYDLAAAYNRVSPAIARAGGYIGCTPRPDDFYKGEGPAVGF
TDHAKTIPVMVGTVFGEFAMMPLPFNKETISEAELDE

>Lipase(2DSN)

SLRANDAPIVLLHGFTGWGREEMFGFKYWGGVRGDIEQWLNDNGYRTYT
LAVGPLSSNWDRACEAYAQLVGGTVDYGAAHAAKHGHARFGRTYPGLLPE
LKRGGRIHIIAHSQGGQTARMLVSLLENGSQEEREYAKAHNVSLSPLFEGGHH
FVLSVTTIATPHDGTTLVNMVDFTDRFFDLQKAVLEAAAVASNVPYTSQVYD
FKLDQWGLRRQPGESFDHYFERLKRSPVWTSTDTARYDLSVSGAEKLNQWV
QASPNTYYLSFSTERTYRGALTGNHYPELGMNAFSAVVCAPFLGSYRNPTLGI
DDRWLENDGIVNTVSMNGPKRGSSDRIVPYDGTLKKGVWNDMGTYNVDHL
EIIGVDPNPSFDIRAFYLRLAEQLASLQP

>Cbotu_EstA(5AH1)

MAEPKAQGTQKVESSTTKKEVKDAEETIKIPTLEDIDNLIDSAEEVKSEEDIN
KMPPLKFPVEFPEVNTRSIIGGNNYPIVLVHGFMGFGRDELLGYKYWGGVVD

LQEKLNASGHETYTATVGPVSSNWDRACELYAYIVGGTVDYGEAHAKKFKH
NRYGRTYPGIYKNISNENKIHLIGHSMGGQTIRTLTQLLSEGSEEEINCGQENIS
PLFEGGKHWIHSVSTISTPNDGTTLSDLMPAKDLISYTFGVLGTITGKNKLFSSI
YDLKLDQWGLKKQNGESQRDYIERVLDSNIWNSTKDIATYDLSTEGAQELNT
WVKAQPDVYYFSWTTQATKESILTGHSVAQIGPMNPIFYPTANLMGRYSRNQ
KDLPIIDKKWFPNDGVVNCISQDGPKLGSNDVIEQYNGGVKIGQWNAMPRIIN
TDHMDIVGTFGNVKDWYMDYASFLSNLSRALEHHHHHH

>Lipase(1JI3)

ASLRANDAPIVLLHGFTGWGREEMFGFKYWGGVRGDIEQWLNDNGYRTY
TLAVGPLSSNWDRVCEAYVQLVGGTVDYGAAHAAKHGHARFGRTYPGLLP
ELKRGGRIHIIAHSQGGQTARMLVSLLENGSQEEREYAKAHNVSLSPLFEGGH
HFVLSVTTIATPHDGTTLVNMVDFTDRFFDLQKAVLEAAAVASNVPYTSQVY
DFKLDQWGLRRQPGESFDHYFERLKRSPVWTSTDTARYDLSVSGAEKLNQW
VQASPNTYYLSFSTERTYRGALTGNHYPELGMNAFSAVVCAPFLGSYRNPTL
GIDSHWLENDGIVNTISMNGPKRGSNDRIVPYDGTLKKGVWNDMGTYNVDH
LEIIGVDPNPSFDIRAFYLRLAEQLASLQP

>Lipase(1HQD)

ADNYAATRYPIILVHGLTGTDKYAGVLEYWYGIQEDLQQRGATVYVANLS
GFQSDDGPNGRGEQLLAYVKTVLAATGATKVNLVGHSQGGLTSRYVAAVA
PDLVASVTTIGTPHRGSEFADFVQGVLAYDPTGLSSTVIAAFVNVFGILTSSSN
NTNQDALAALKTLTTAQAATYNQNYPSAGLGAPGSCQTGAPTETVGGNTHL
LYSWAGTAIQPTISVFGVTGATDTSTIPLVDPANALDPSTLALFGTGTVMVNR
GSGQNDGVVSKCSALYGQVLSTSYKWNHLDEINQLLGVRGANAEDPVAVIR
THANRLKLAGV

>Lipase (1EX9)

STYTQTKYPIVLAHGMLGFDNILGVDYWFGIPSALRRDGAQVYYVTEVSQLD
TSEVRGEQLLQQVEEIVALSGQPKVNLIGHSHGGPTIRYVAAVRPDLIASATS
VGAPHKGSDTADFLRQIPPGSAGEAVLSGLVNSLGALISFLSSGSTGTQNSLGS
LESLNSEGAARFNAKYPQGIPTSACGEGAYKVNGVSYYSWSGSSPLTNFLDPS
DAFLGASSLTFKNGTANDGLVGTCSSHLGMVIRDNYRMNHLDEVNQVFGLT
SLFETSPVSVYRQHANRLKNASL

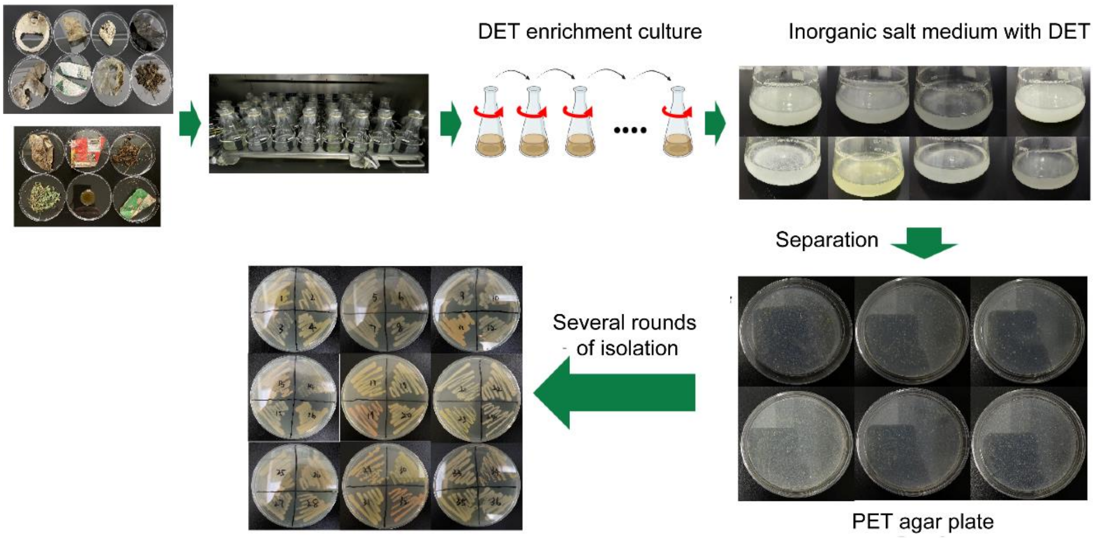

Supplementary Fig. 1 Screening and purification of the strains that capable of growing on PET powder plate. Reaction conditions: pH 7.5, 30 °C, 2 g/L PET powder, and 5% inoculum for 5 days.

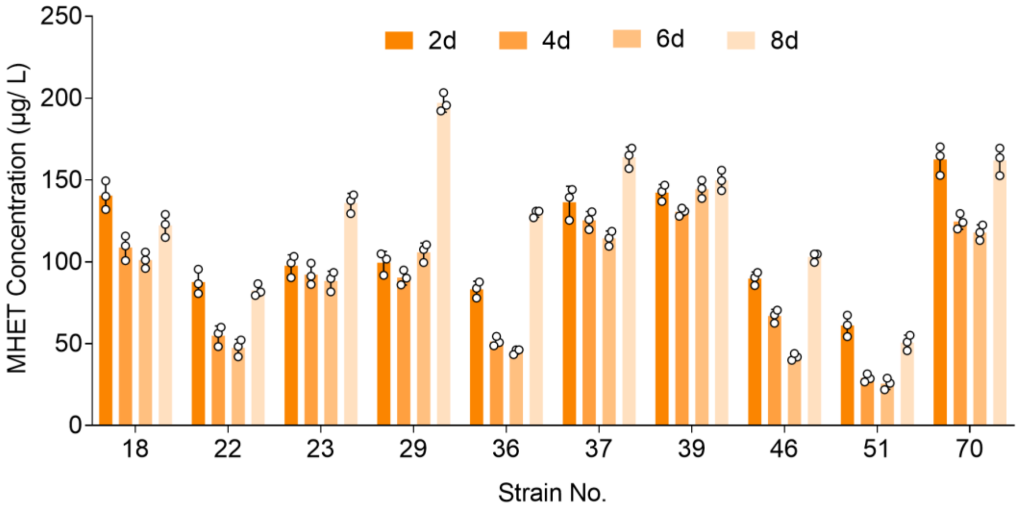

Supplementary Fig. 2 The performance of ten microbes with MHET concentration over 55 μg/L during degrading PET film. Reaction conditions: pH 7.5, 30 °C, 2 g/L PET powder, and 5% inoculum for 8 days. Error bars correspond to the standard deviation (s.d.) of three measurements (n=3). Source data are provided as a Source Data file.

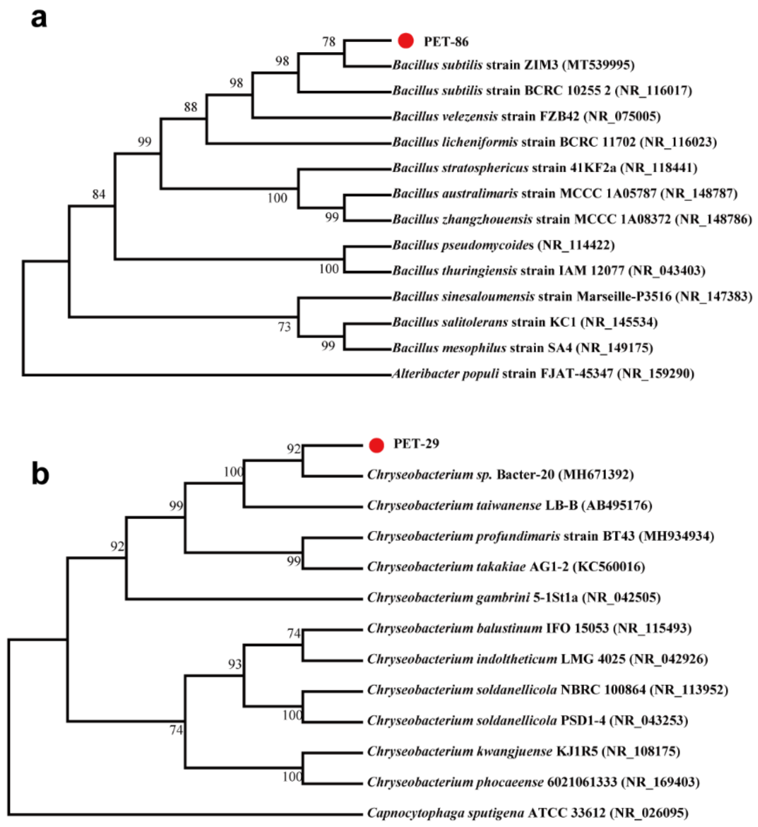

Supplementary Fig. 3 Phylogenetic tree based on 16S rRNA gene sequences. (a) *Bacillus subtilis* PET-86 (No.70) and (b) *Chryseobacterium sp*. PET-29 (No.29)

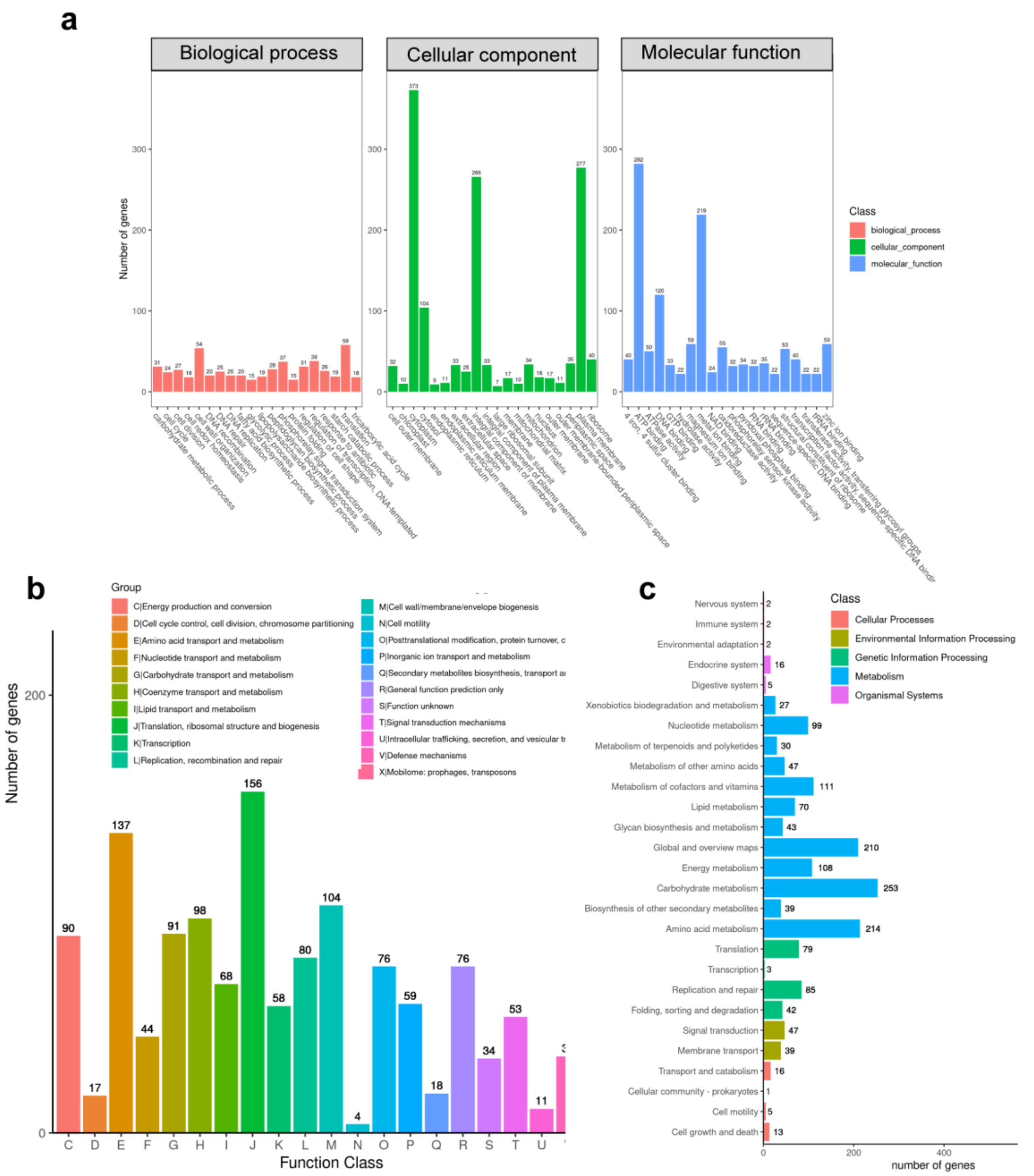

Supplementary Fig. 4 Genome analysis of *Chryseobacterium sp.* PET-29. (a) All genes biological processes by GO enrichment analysis. (b) Genome functional annotation of *Chryseobacterium sp.* PET-29 chromosome against the COG database. (c) The pathway classification of *Chryseobacterium sp.* PET-29 chromosome against the KEGG database.

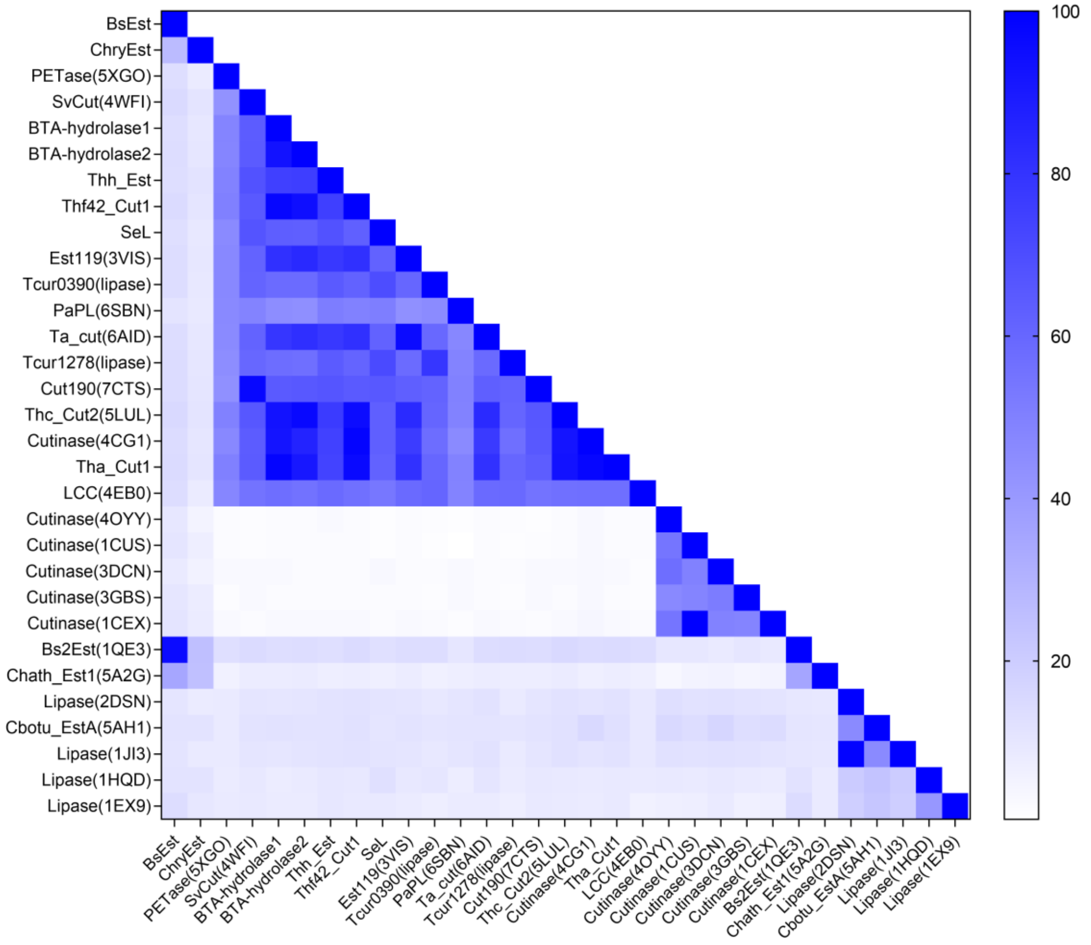

Supplementary Fig. 5 Sequence identity results of ChryBHETase with other reported PET enzymes. Source data are provided as a Source Data file.

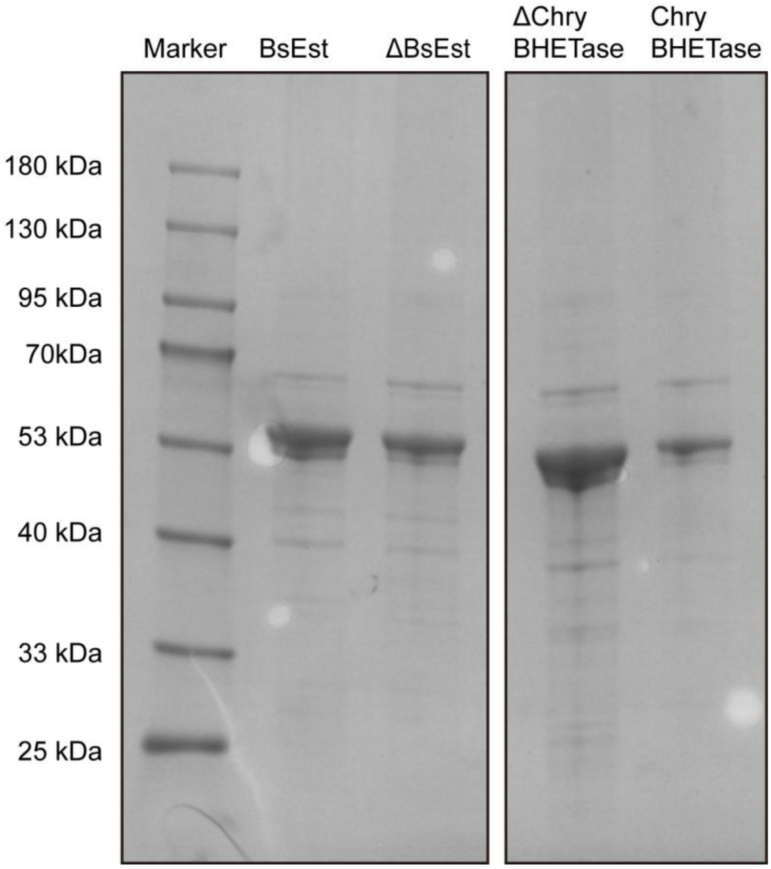

Supplementary Fig. 6 Heterologous expression of BsEst, ΔBsEst, ChryBHETase, and ΔChryBHETase mediated by PelB Signal peptides and Protein purification. Samples were run on the same gel, with the same vehicle sample, and image was cropped at the black line only for the purpose of this figure. Results were reproduced three times independently; representative gels are shown. Source data are provided as a Source Data file.

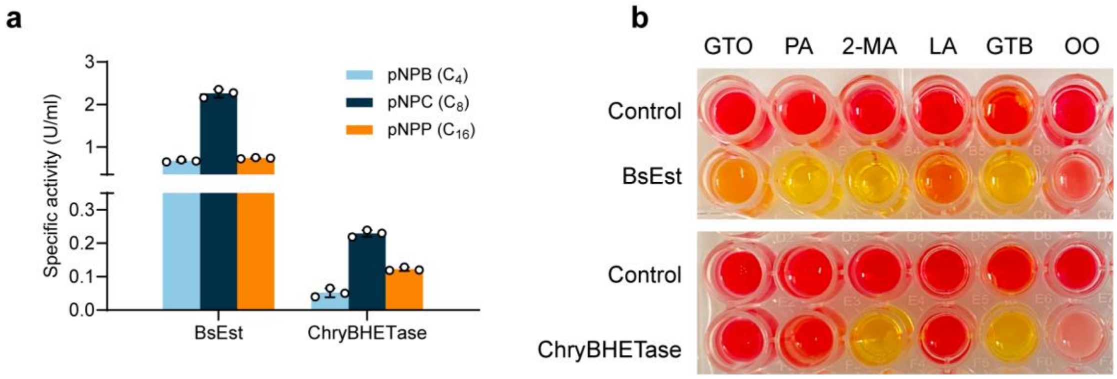

Supplementary Fig. 7 The substrate specificity of BsEst and ChryBHETase. (a) Specific activity of BsEst and ChryBHETase with different carbon chain lengths of *p*NPB (C_4), *p*NPC (C_8), and *p*NPP (C_{12}) as substrate. Reaction condition: the crude enzymes were incubated with *p*NPB, *p*NPC and *p*NPP in a buffer containing 50 mM Na_2HPO_4-NaH_2PO_4 (pH 7.5) at 40 °C. Error bars correspond to the standard deviation (s.d.) of three measurements (n=3). (b) Substrate specificity of BsEst and ChryBHETase for other phenyl esters, glycerides, and tertiary alcohols. Abbreviation：GTO: Glyceryl trioctanoate; PA: Phenyl acetate; 2-MA: 2-Methylbutyl acetate; LA: Linalyl acetate; GTB: Glyceryl tributyrate; OO: Olive oil. The deeper the yellow, the greater the hydrolysis capacity. Source data are provided as a Source Data file.

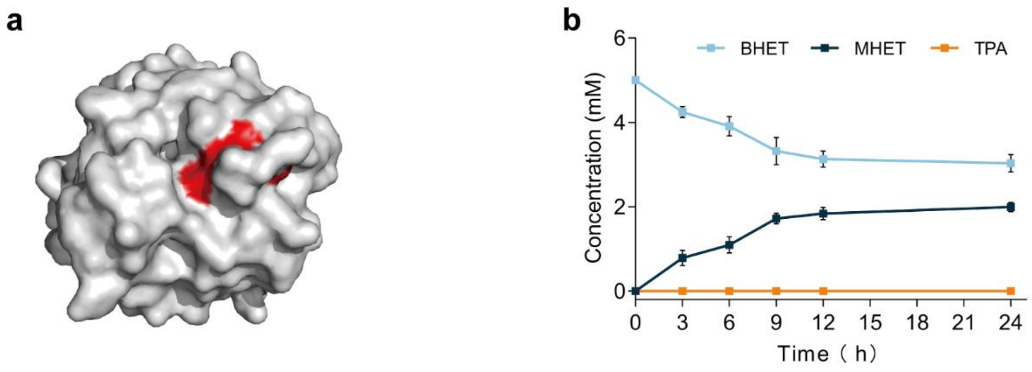

Supplementary Fig. 8 The activities of BSLA upon BHET. (a) 3D structure of BSLA. Red: the catalytic triad of BSLA. (b) The activities of BSLA upon BHET. Reaction condition: 5 mM BHET at 30 °C in pH 7.5 50 mM Na_2HPO_4-NaH_2PO_4 buffer for 24 h. Error bars correspond to the standard deviation (s.d.) of three measurements (n=3). Source data are provided as a Source Data file.

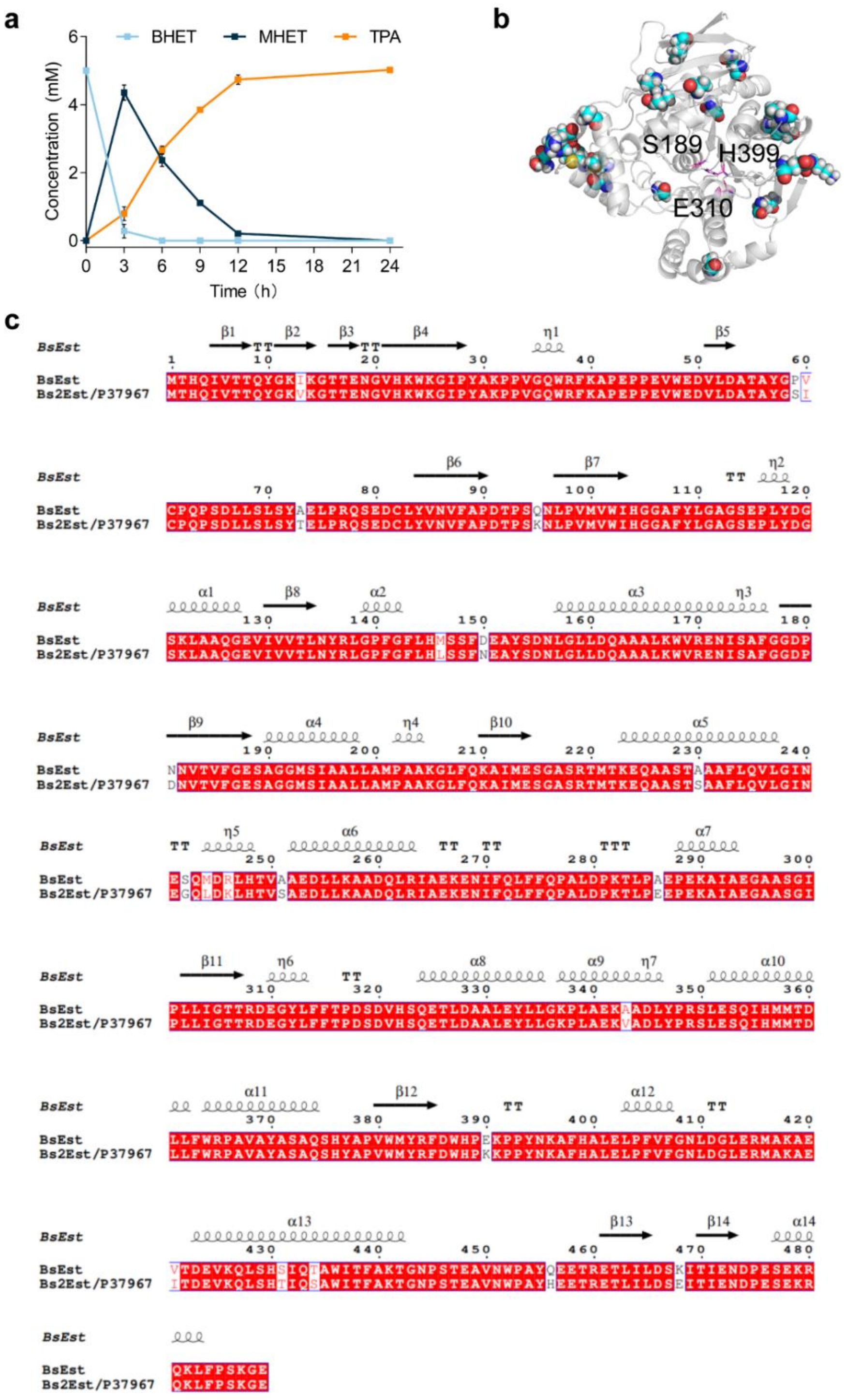

Supplementary Fig. 9 The comparison of BsEst and Bs2Est. (a) The activities of BsEst and Bs2Est (UniProtKB Accession: P37967) upon BHET. Reaction condition: 5 mM BHET at 30 °C in pH 7.5 buffer for 24 h. Error bars correspond to the standard deviation (s.d.) of three measurements (n=3). Source data are provided as a Source Data file. (b) Differences in 3D structure between BsEst and Bs2Est. (c) Sequence alignment of BsEst and Bs2Est (95.7% amino acid identity).

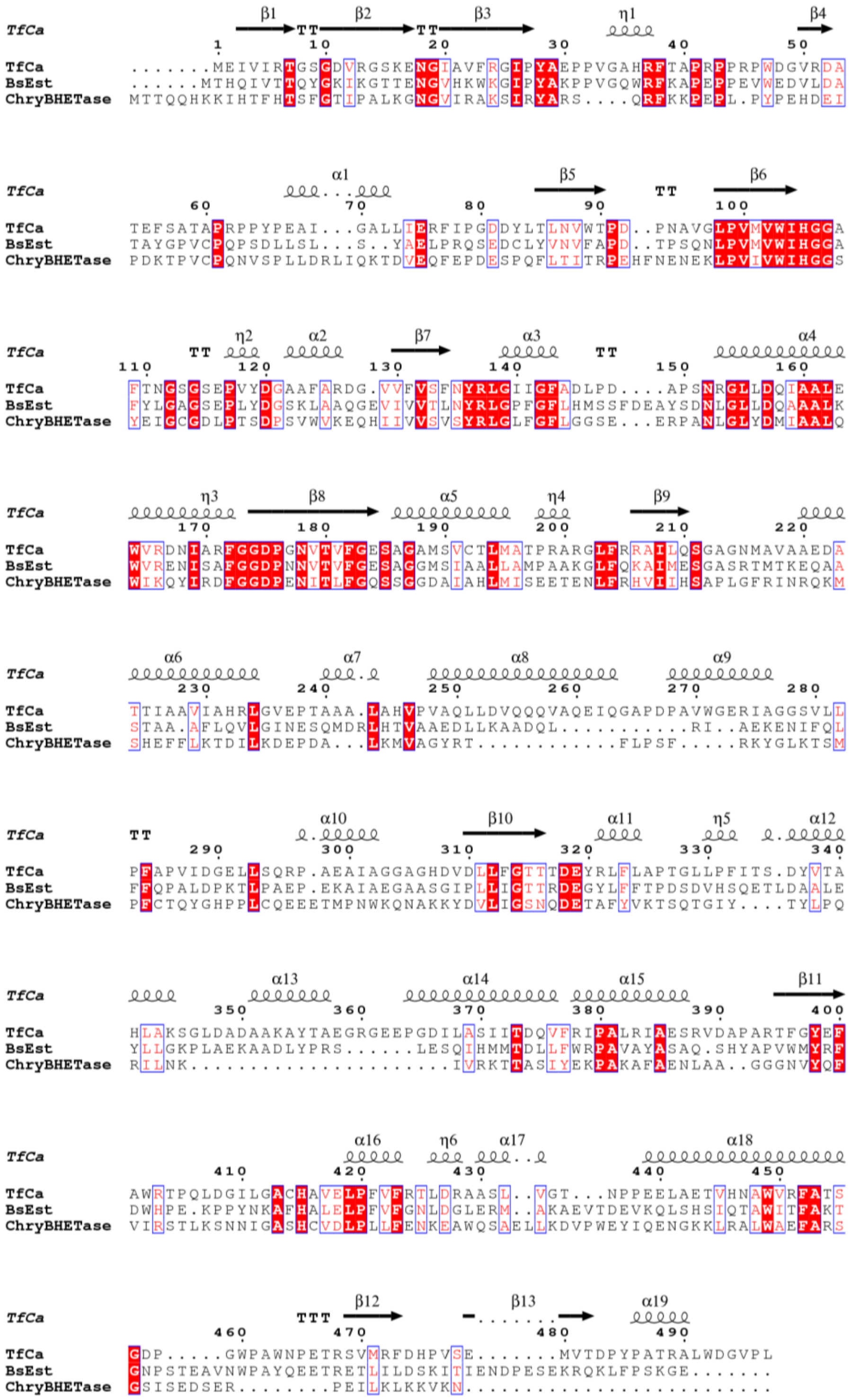

Supplementary Fig. 10 The sequence alignment of TfCa, BsEst and ChryBHETase. Source data are provided as a Source Data file.

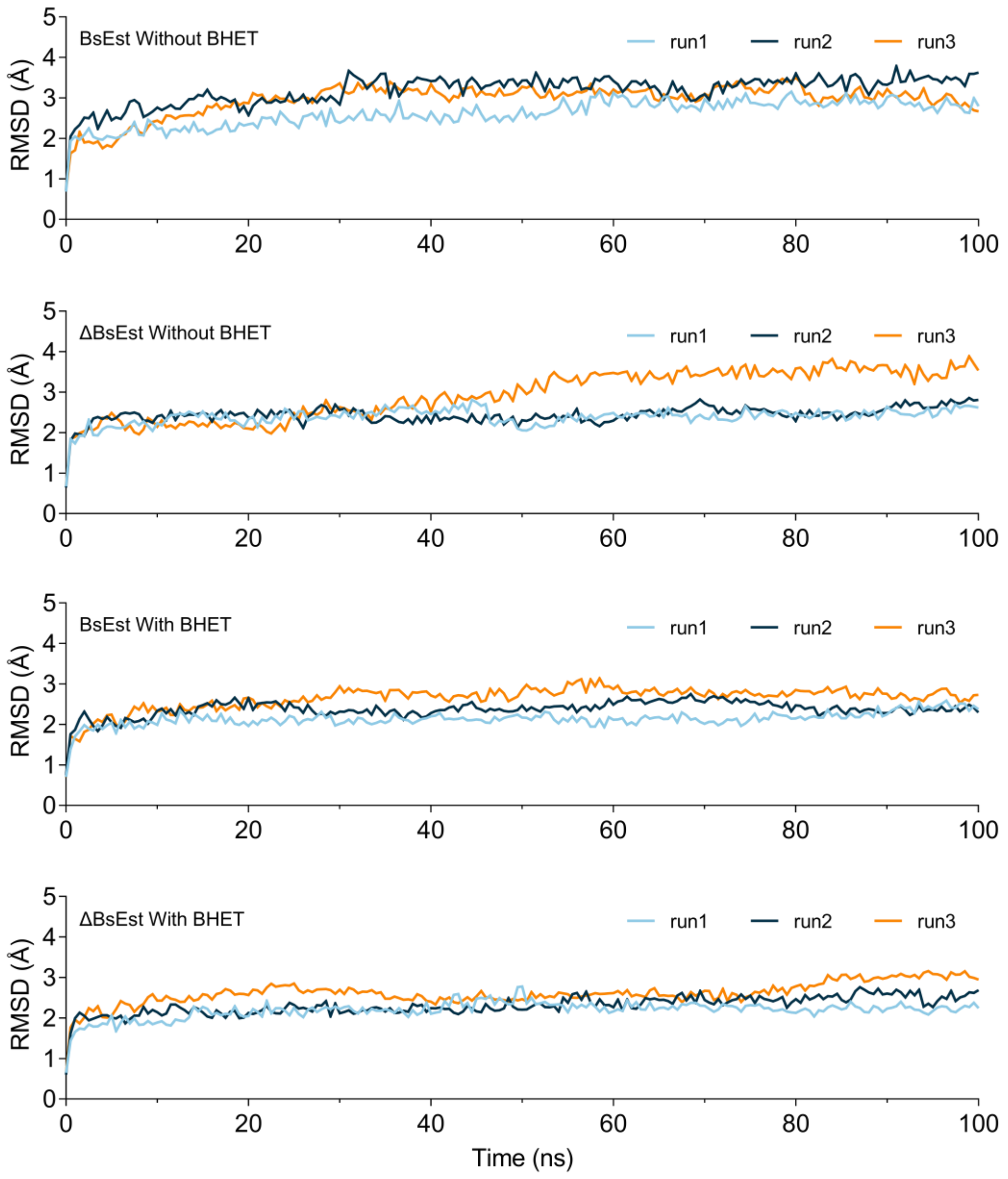

Supplementary Fig. 11 Root mean square deviation (RMSD) of BsEst and ΔBsEst backbone with respect to the initial structure as a function of time with/without BHET. Three independent MD runs for each simulation are shown (n=3). Source data are provided as a Source Data file.

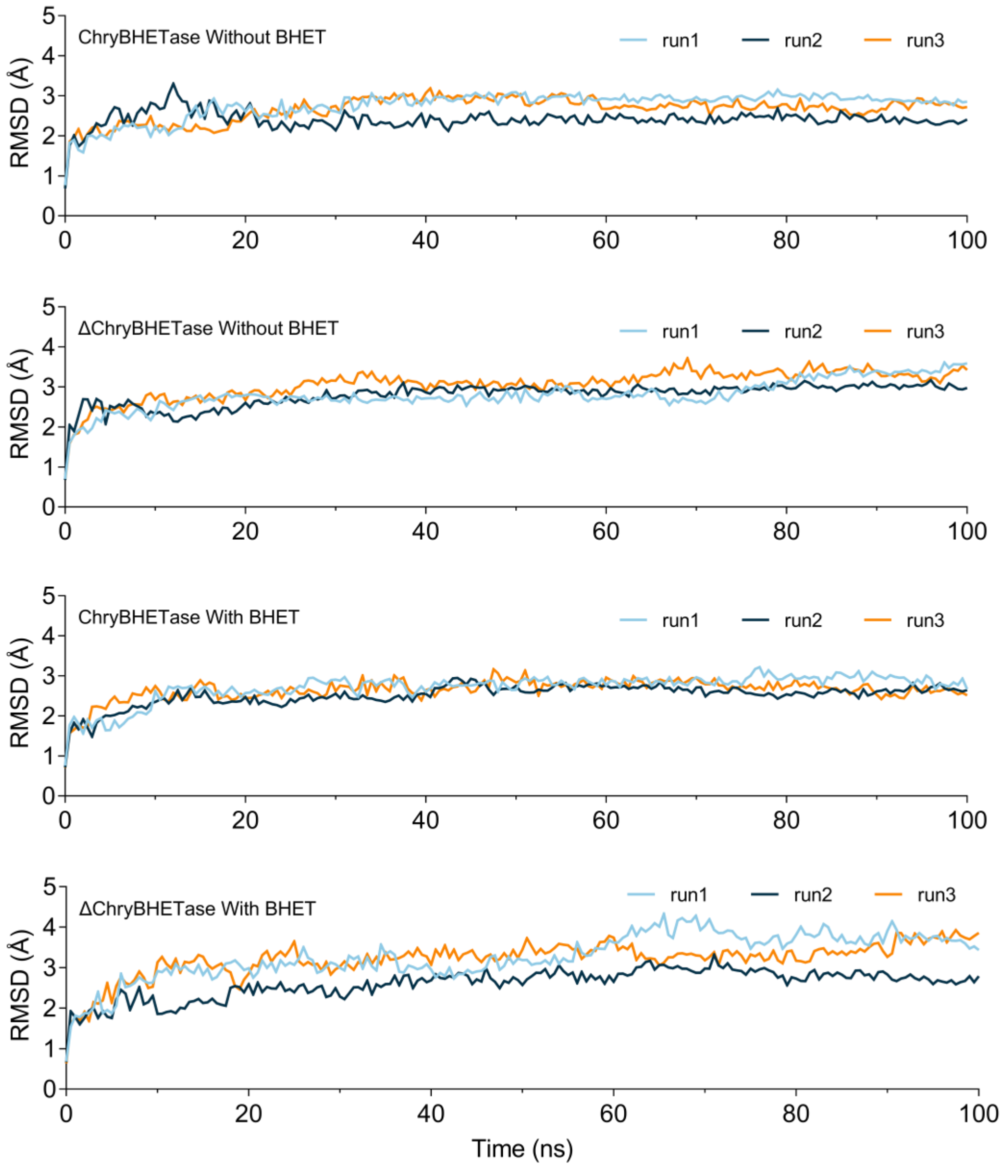

Supplementary Fig. 12 Root mean square deviation (RMSD) of ChryBHETase and ΔChryBHETase backbone with respect to the initial structure as a function of time with/without BHET. Three independent MD runs for each simulation are shown (n=3). Source data are provided as a Source Data file.

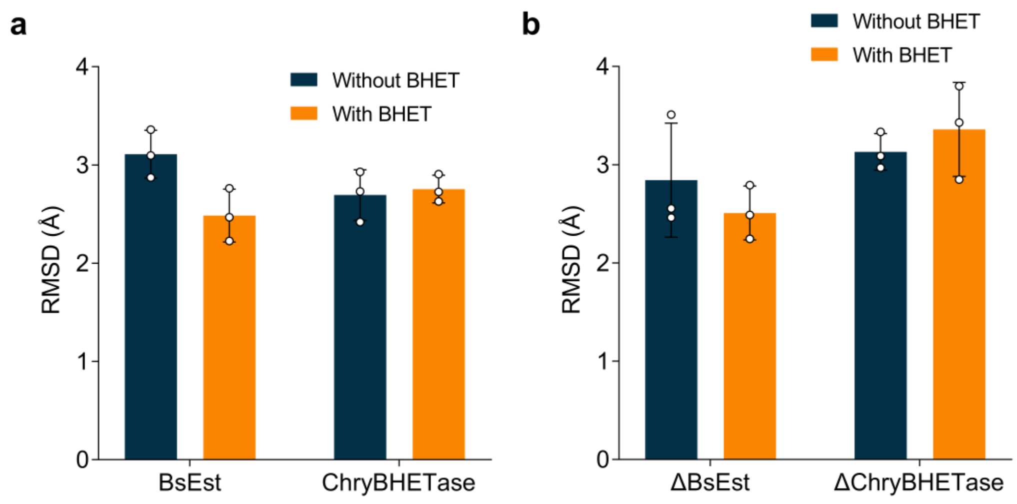

Supplementary Fig. 13 Time-average RMSD of (a) BsEst and ChryBHETase and (b) ΔBsEst and ΔChryBHETase determined from the last 40 ns with/without BHET. Error bars correspond to the standard deviation (s.d.) of three independent MD runs (n=3). Source data are provided as a Source Data file.

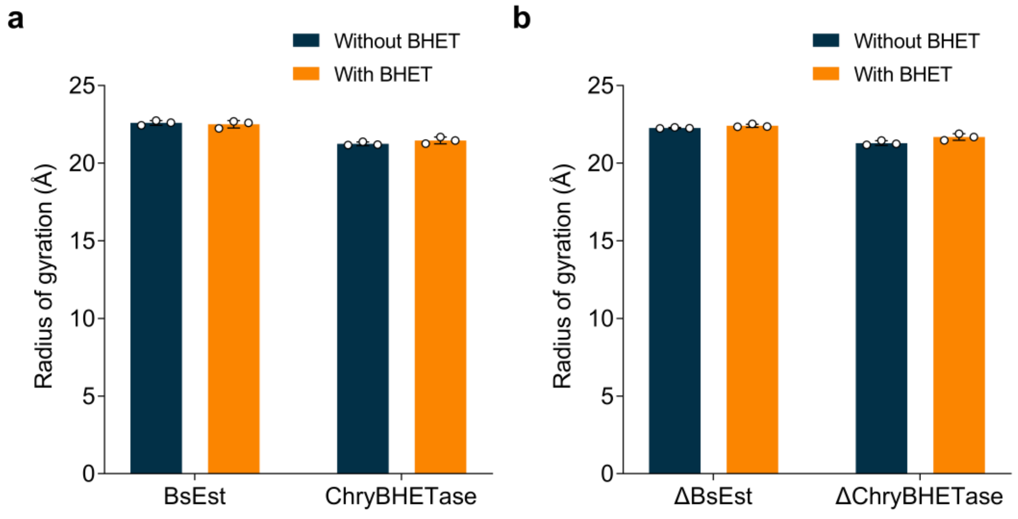

Supplementary Fig. 14 The radius of gyration (R_g) of (a) BsEst and ChryBHETase and (b) ΔBsEst and ΔChryBHETase determined from the last 40 ns with/without BHET. Error bars correspond to the standard deviation (s.d.) of three independent MD runs (n=3). Source data are provided as a Source Data file.

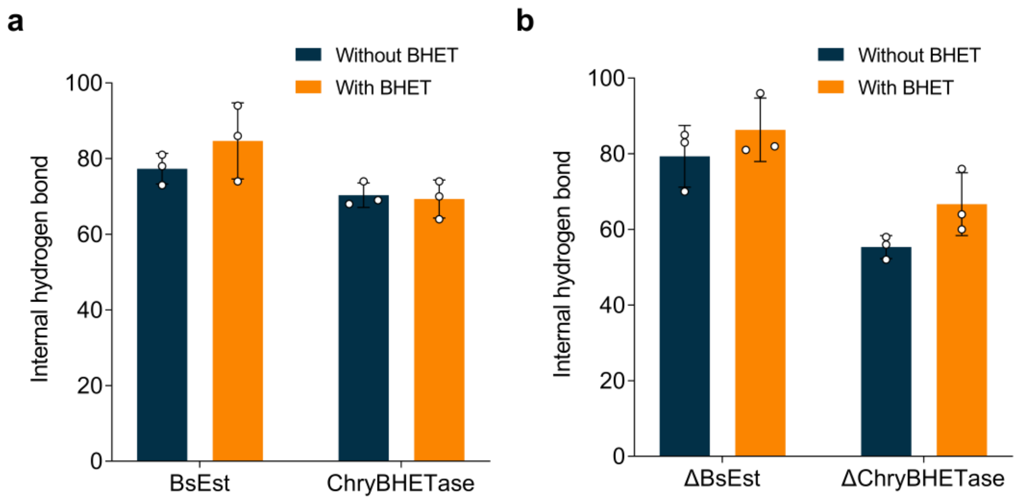

Supplementary Fig. 15 The number of internal hydrogen bonds of (a) BsEst and ChryBHETase and (b) ΔBsEst and ΔChryBHETase with > 95 % occupancy determined from the last 40 ns with/without BHET.Error bars correspond to the standard deviation (s.d.) of three independent MD runs (n=3). Source data are provided as a Source Data file.

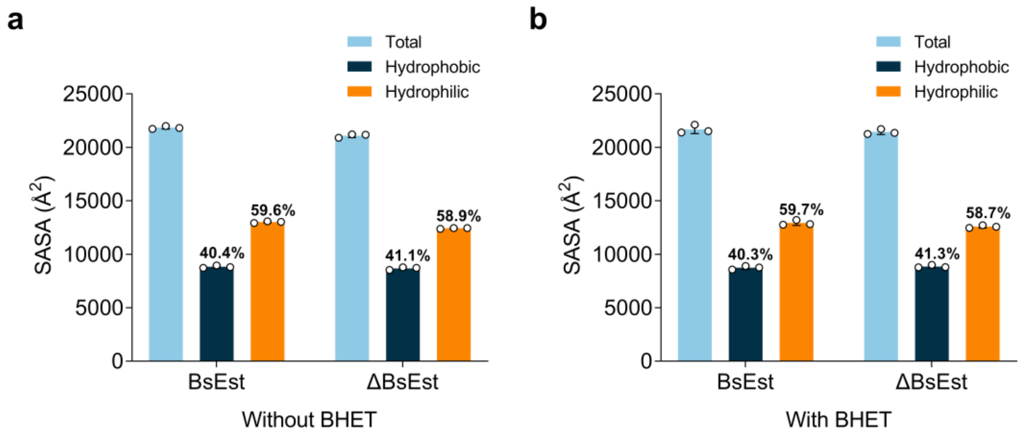

Supplementary Fig. 16 The time-averaged total SASA, hydrophobic SASA, and hydrophilic SASA of BsEst and ΔBsEst (a) without BHET and (b) with BHET determined from the last 40 ns. The percentage of hydrophobicity increased after the truncation of the enzyme. Error bars correspond to the standard deviation (s.d.) of three independent MD runs (n=3). Source data are provided as a Source Data file.

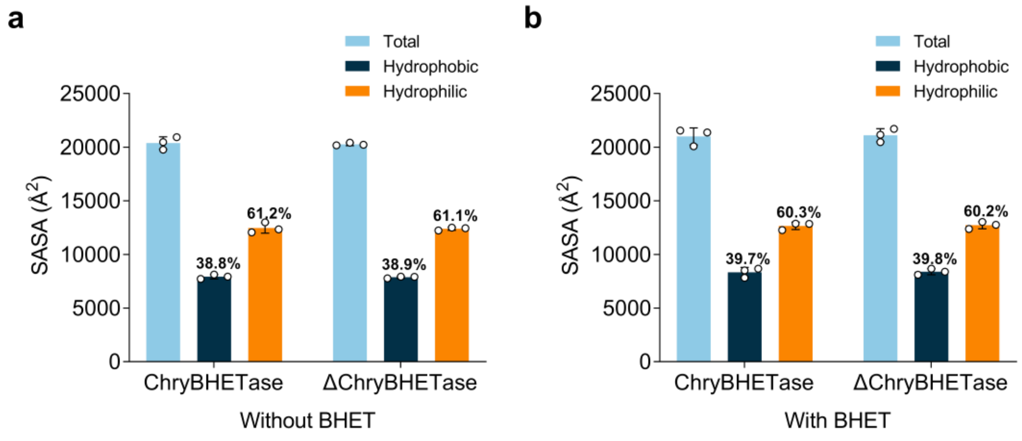

Supplementary Fig. 17 The time-averaged total SASA, hydrophobic SASA, and hydrophilic SASA of ChryBHETase and ΔChryBHETase (a) without BHET and (b) with BHET determined from the last 40 ns. The percentage of hydrophobicity increased after the truncation of the enzyme. Error bars correspond to the standard deviation (s.d.) of three independent MD runs (n=3). Source data are provided as a Source Data file.

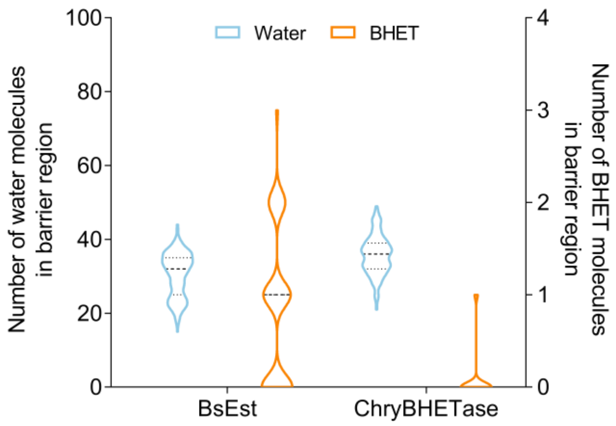

Supplementary Fig. 18 The number of BHET/water molecules in barrier region of BsEst and ChryBHETase. The dotted line represents the quartiles of the data and the middle dotted line represents the median. Data plotted from last 40 ns of three independent MD runs (n=243). Source data are provided as a Source Data file.

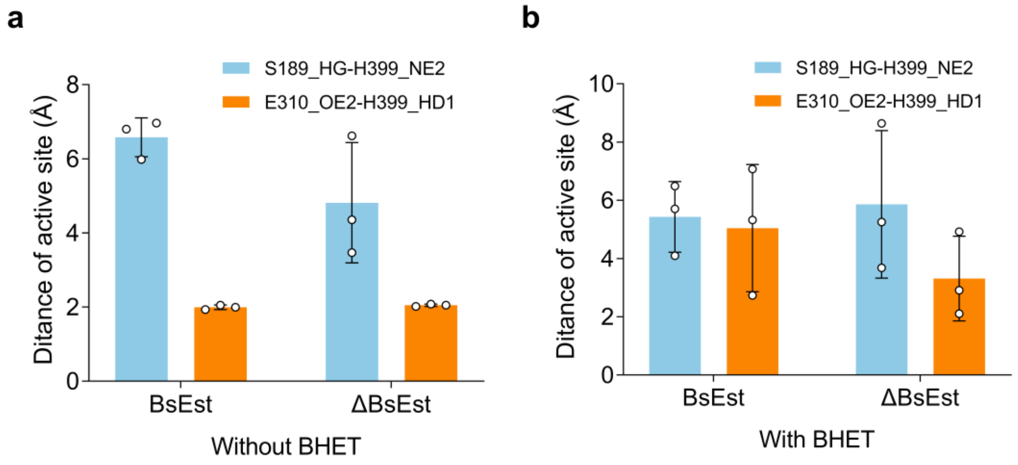

Supplementary Fig. 19 The distance of BsEst and ΔBsEst active site (a) without BHET and (b) with BHET. Light blue: the distance between the HG atom of Serine 189 and the NE2 atom of Histidine 399; Orange: the distance between the HD1 atom of Histidine 399 and the OE2 atom of Glutamic acid 310. Error bars correspond to the standard deviation (s.d.) of three independent MD runs (n=3). Source data are provided as a Source Data file.

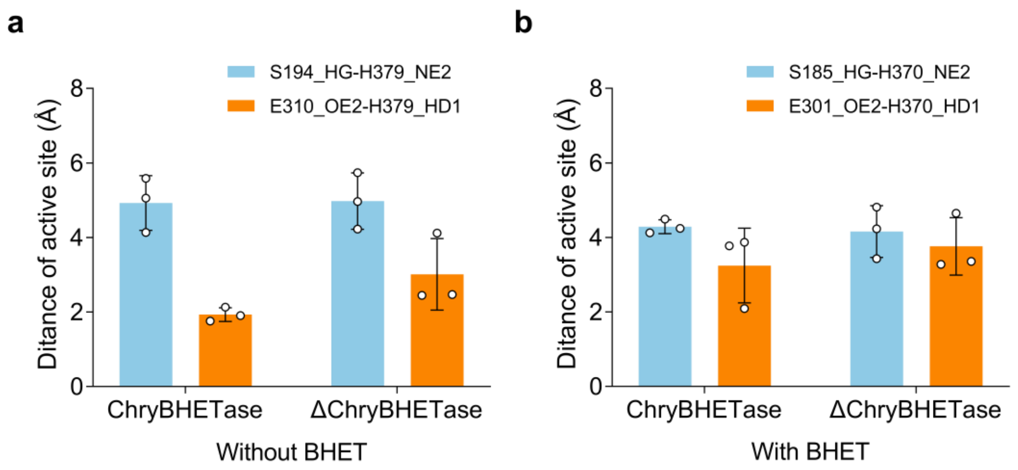

Supplementary Fig. 20 The distance of ChryBHETase and ΔChryBHETase active site (a) without BHET and (b) with BHET. Light blue: the distance between HG atom of Serine 194 and the NE2 atom of histidine 379; Orange: the distance between the OE2 atom of Glutamic acid 310 and the HD1 atom of histidine 379. Error bars correspond to the standard deviation (s.d.) of three independent MD runs (n=3). Source data are provided as a Source Data file.

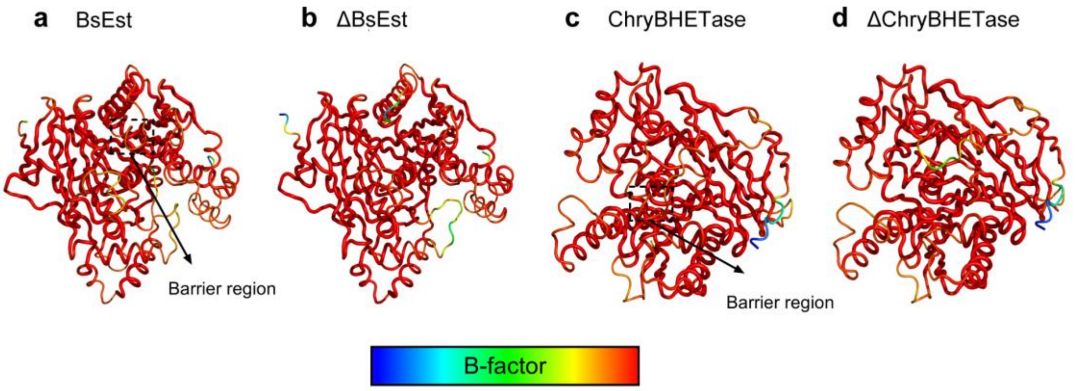

Supplementary Fig. 21 The B-factor of the optimal ΔBHETase and BHETase. (a) BsEst, (b) ΔBsEst. (c) ChryBHETase and (d) ΔChryBHETase. Low B-factor in blue, high B-factor in red. The closer to red, the greater the flexibility.

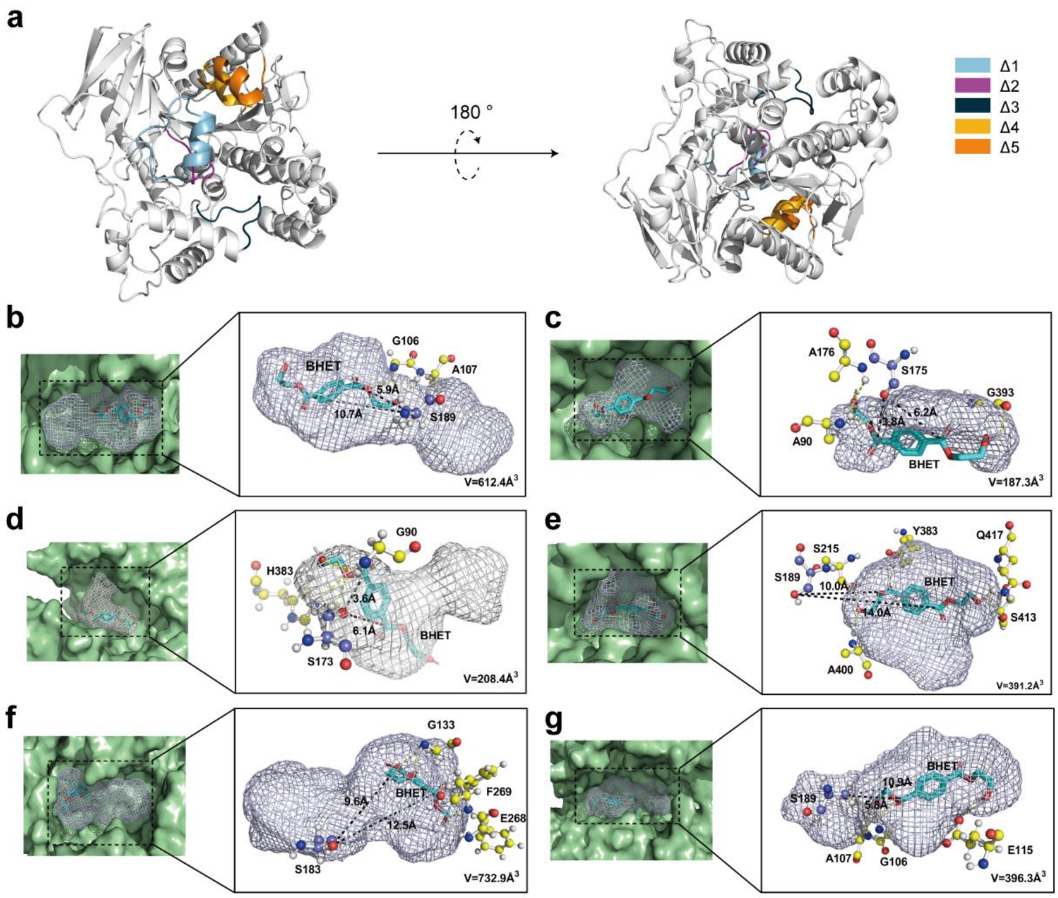

Supplementary Fig. 22 Binding mode analysis of BsEst and its truncated variants with BHET. (a) The overall structure of BsEst with five truncated sites. (b) Binding mode analysis of wide type BsEst. Five truncated variants were generated by deleting (c) V60-R77 (BsEst-Δ1), (d) G105-A112 (BsEst-Δ2), (e) K267-F275 (BsEst-Δ3), (f) L401-K418 (BsEst-Δ4) and (g) L410-K418 (BsEst-Δ5), respectively. Purple: the active sites. Yellow: the surrounding amino acids that form hydrogen bonds with BHET. BHET is colored with cyan stick.

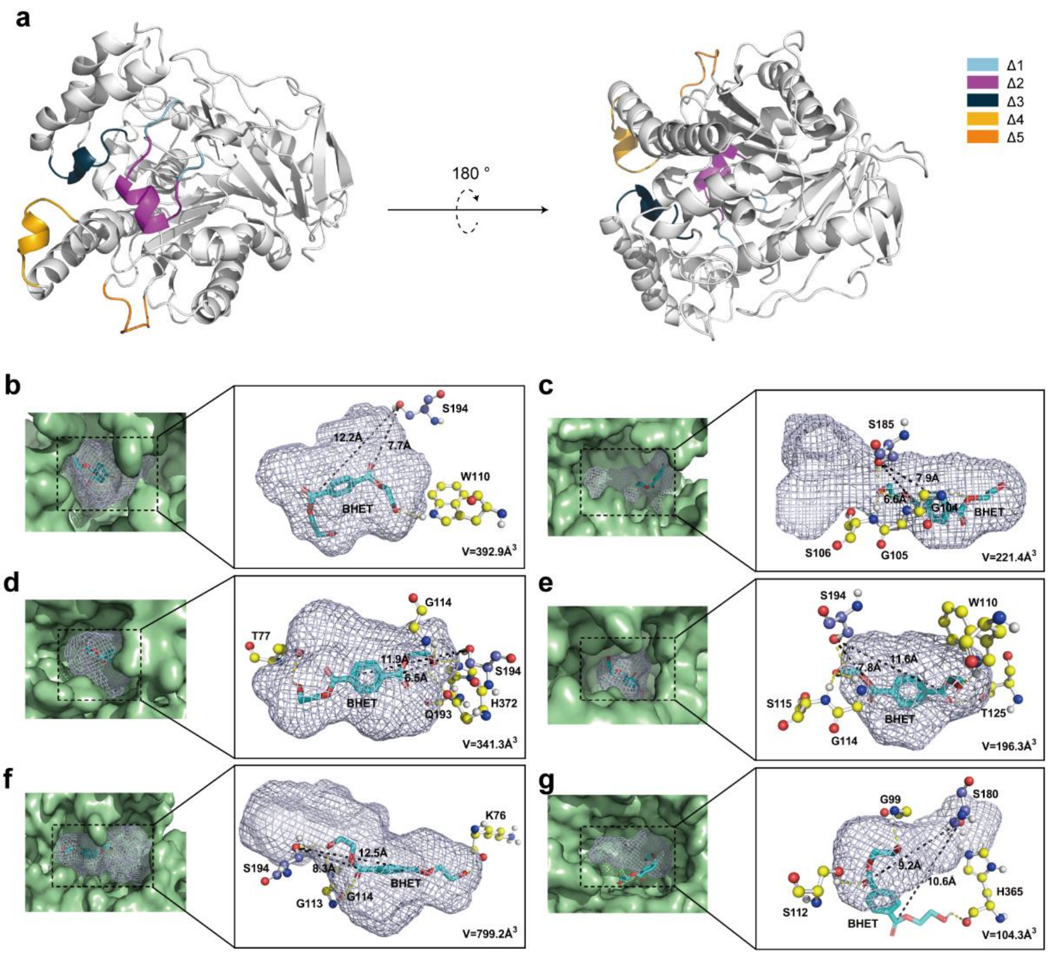

Supplementary Fig. 23 Binding mode analysis of ChryBHETase and its truncated variants with BHET. (a) The overall structure of ChryBHETase with five truncated sites. (b) Binding mode analysis of wide type ChryBHETase. Five truncated variants were generated by deleting (c) P63-D78 (ChryBHETase-Δ1), (d) V66-K76 (ChryBHETase-Δ2), (e) G267-F274 (ChryBHETase-Δ3), (f) Q319-P327 (ChryBHETase-Δ4) and (g) S368-G376 (ChryBHETase-Δ5), respectively. Purple: the active sites. Yellow: the surrounding amino acids that form hydrogen bonds with BHET. BHET is colored with cyan stick.

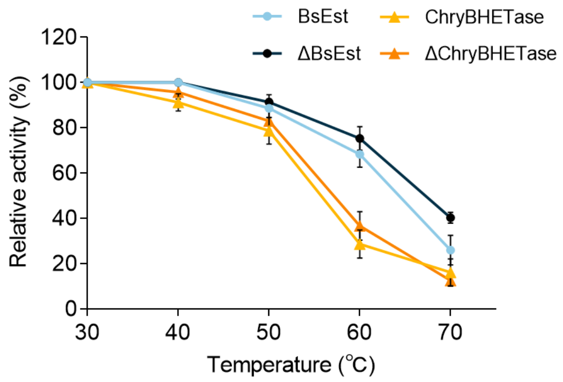

Supplementary Fig. 24 Thermostability of ΔBsEst, ΔChryBHETase and their wide type from 30 °C to 70 °C. The residual activities were measured after 2 h of incubation and displayed as a relative percentage of their corresponding initial activities. Error bars correspond to the standard deviation (s.d.) of three independent MD runs (n=3). Source data are provided as a Source Data file.

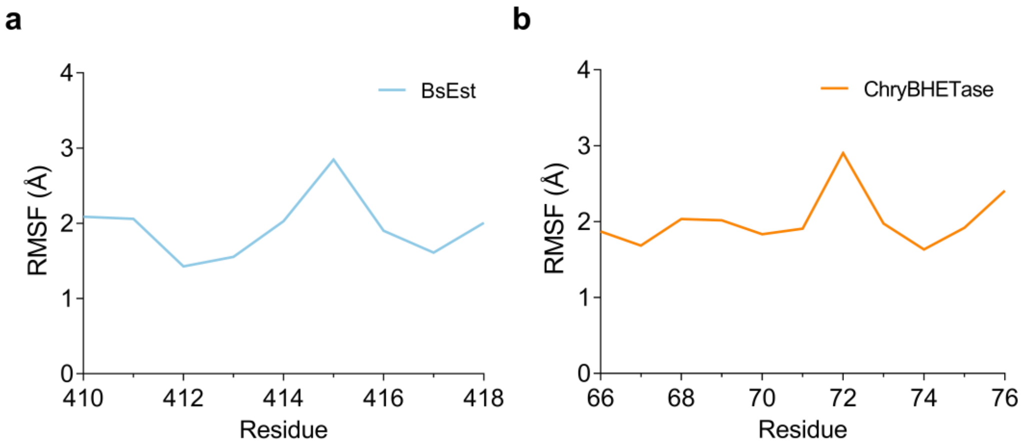

Supplementary Fig. 25 RMSF of (a) BsEst truncated region (residue L410 to K418) and (b) ChryBHETase truncated region (residue V66 to K76). Data plotted from the average of three independent MD runs (n=3). Source data are provided as a Source Data file.

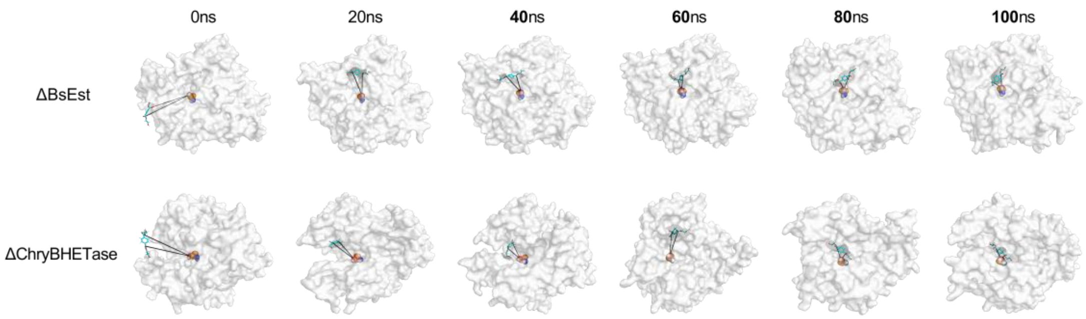

Supplementary Fig. 26 Changes in the position of overall structure of ΔBsEst and ΔChryBHETase between the active sites and BHET from 0-100 ns. The active site was shown to spheres, and the BHET molecule was shown to sticks.

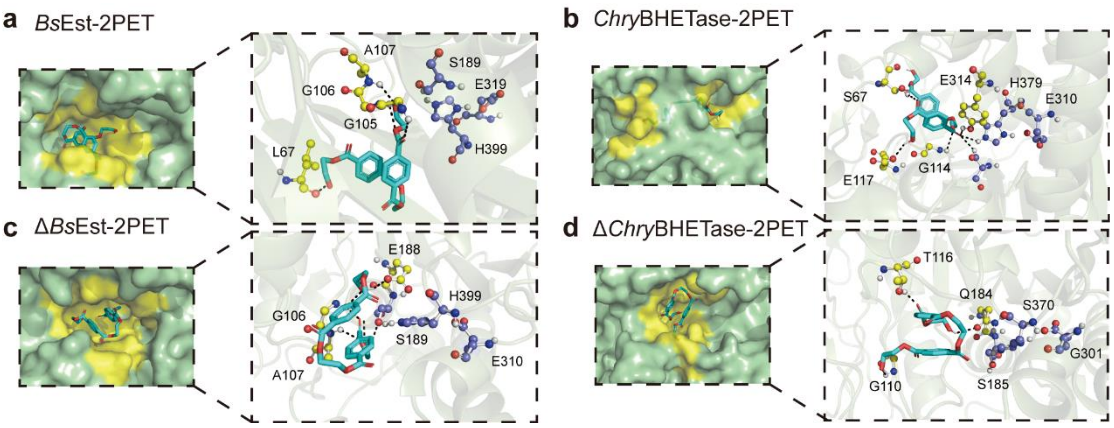

Supplementary Fig. 27 Binding mode analysis of ΔBsEst, ΔChryBHETase and their wide type with 2PET. (a) BsEst; (b) ΔBsEst; (c) ChryBHETase, and (d) ΔChryBHETase. Yellow: the surrounding residues with hydrogen bonding to 2PET. Purple: the catalytic triad. Black dashed line: hydrogen bonding forces.

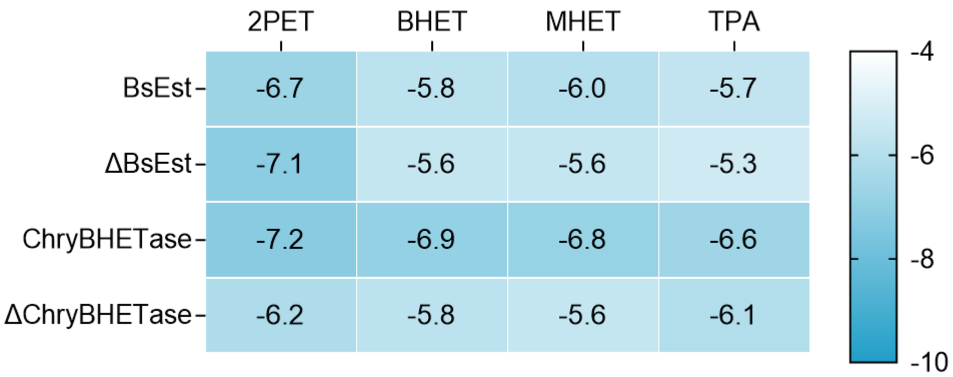

Supplementary Fig. 28 The binding energy results of different ligands (2PET, BHET, MHET, and TPA) with ΔBsEst, BsEst, ΔChryBHETase and ChryBHETase in molecular docking.

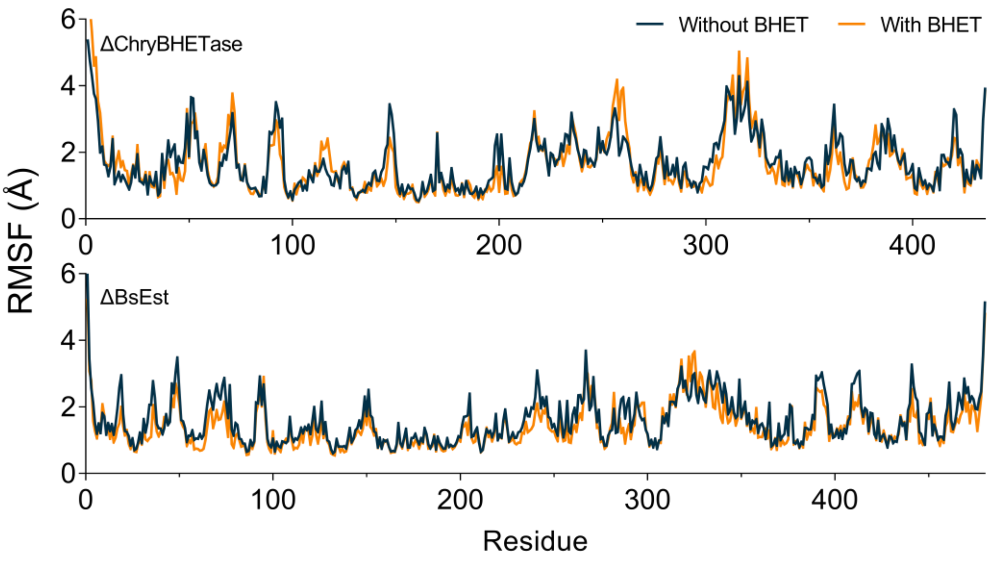

Supplementary Fig. 29 RMSF of ΔBsEst and ΔChryBHETase residues determined from the last 40 ns of MD with/without BHET. Data plotted from the average of three independent MD runs. Data plotted from the average of three independent MD runs (n=3). Source data are provided as a Source Data file.

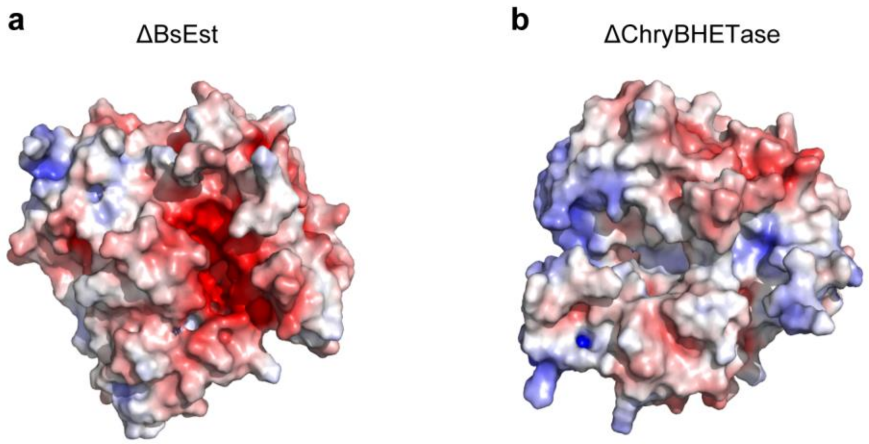

Supplementary Fig. 30 Electrostatic potential energy distribution of (a) ΔBsEst and (b) ΔChryBHETase. The positive charge distribution is shown in blue. The negative charge distribution is shown in red and white in the neutral distribution. The regions within the dashed boxes are the truncated regions of BsEst and ChryBHETase. The electrostatic potential energy ranges from -5 to 5 kcal/mol with red to blue.

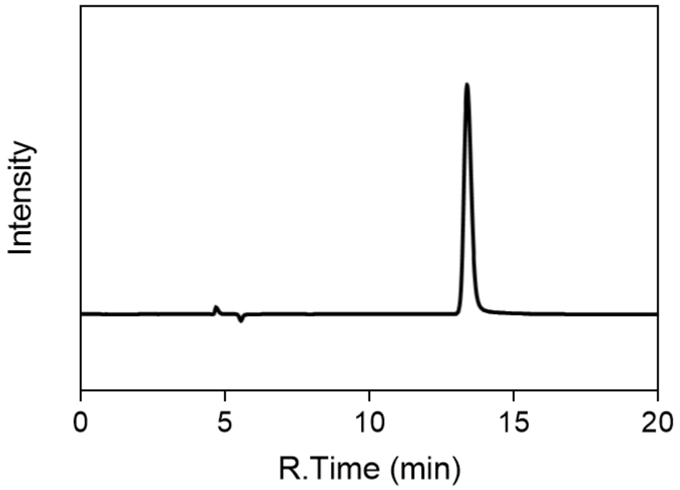

Supplementary Fig. 31 High Performance Liquid Chromatography (HPLC) data of homogeneous TPA. Source data are provided as a Source Data file.

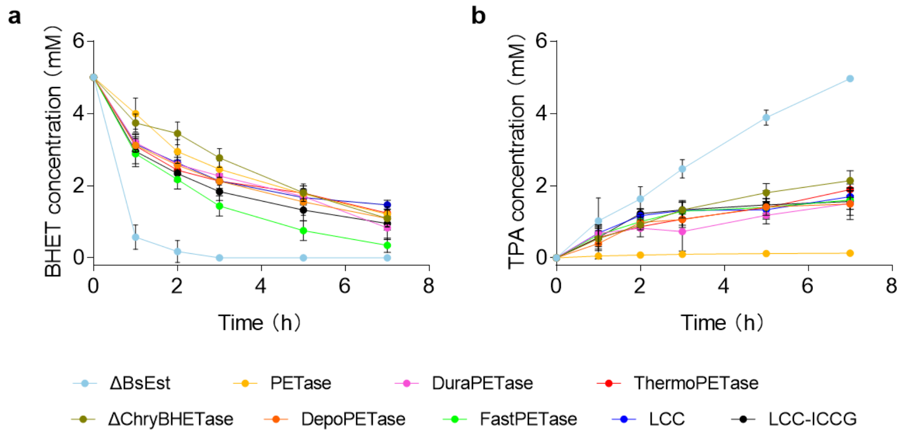

Supplementary Fig. 32 The reaction curve of ΔBHETases and other PET hydrolases. (a) Depletion curve of BHET as a substrate. (b) Generation curve of TPA as a terminal product. Reaction condition: 5 mM BHET and 0.5mg/mL ΔBHETases at 60 °C in pH 8.5 buffer for 7 h. TPA conversion rate of ΔBsEst is 31.6 mM/h/mg_{enzyme}, ΔChryBHETase is 14.4 mM/h/mg_{enzyme}, DepoPETase is 10.2 mM/h/mg_{enzyme}, FAST-PETase is 10.46 mM/h/mg_{enzyme}, DuraPETase is 8.56 mM/h/mg_{enzyme}, ThermoPETase is 10.44 mM/h/mg_{enzyme}, LCC is 9.56 mM/h/mg_{enzyme}, and LCC-ICCG is 10.12 mM/h/mg_{enzyme}. Error bars correspond to the standard deviation (s.d.) of three measurements (n=3). Source data are provided as a Source Data file.

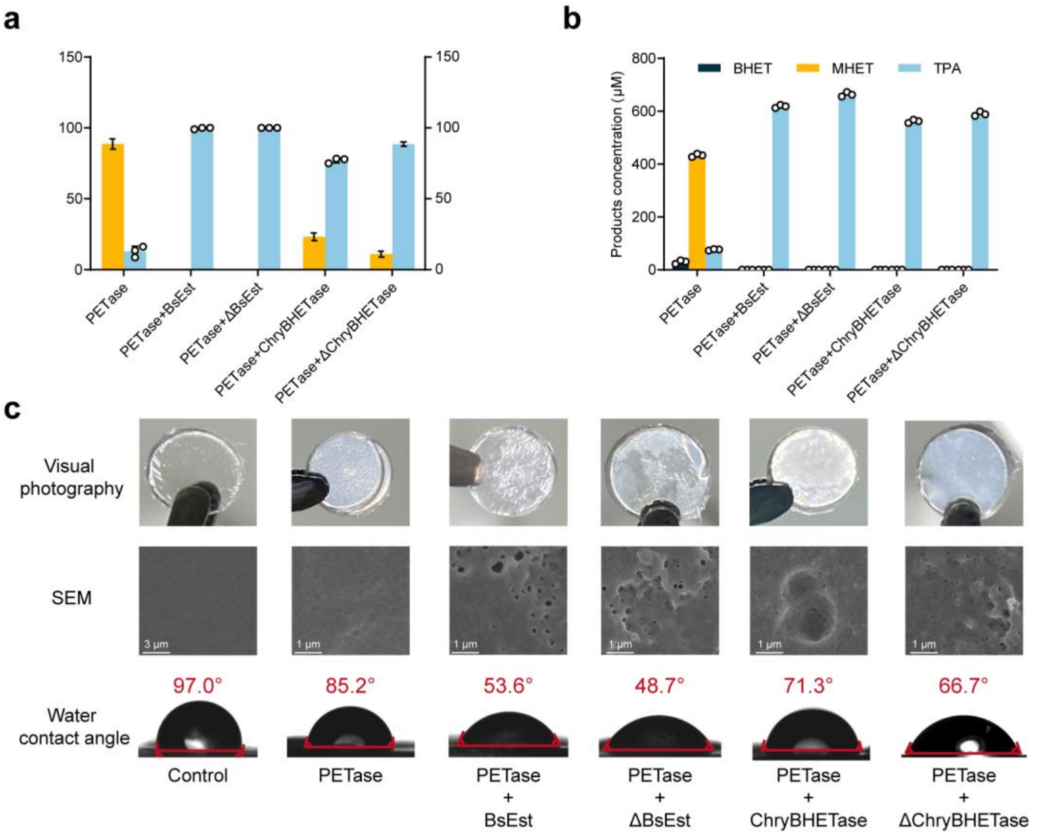

Supplementary Fig. 33 Two-enzyme degradation system consisting of PETase and ΔBHETase. The biodegradation performance of PETase and two-enzyme system towards **(a)** BHET and **(b)** PET film. Reaction condition: 5 mM BHET or PET films (ø=6 mm) were soaked in 2940 μL of Na_2HPO_4-NaH_2PO_4 (pH 8.5, 50 mM) buffer at 30 °C with 50 μL of 0.5 mg/mL PET hydrolases and ΔBHETases for 24 h, respectively. Error bars correspond to the standard deviation (s.d.) of three measurements **(c)** The visual photography (up panel) and SEM images (middle panel), and water contact angle analysis of the PET film after biodegradation. Results were reproduced three times independently; representative photography and micrographs are shown. Source data are provided as a Source Data file.

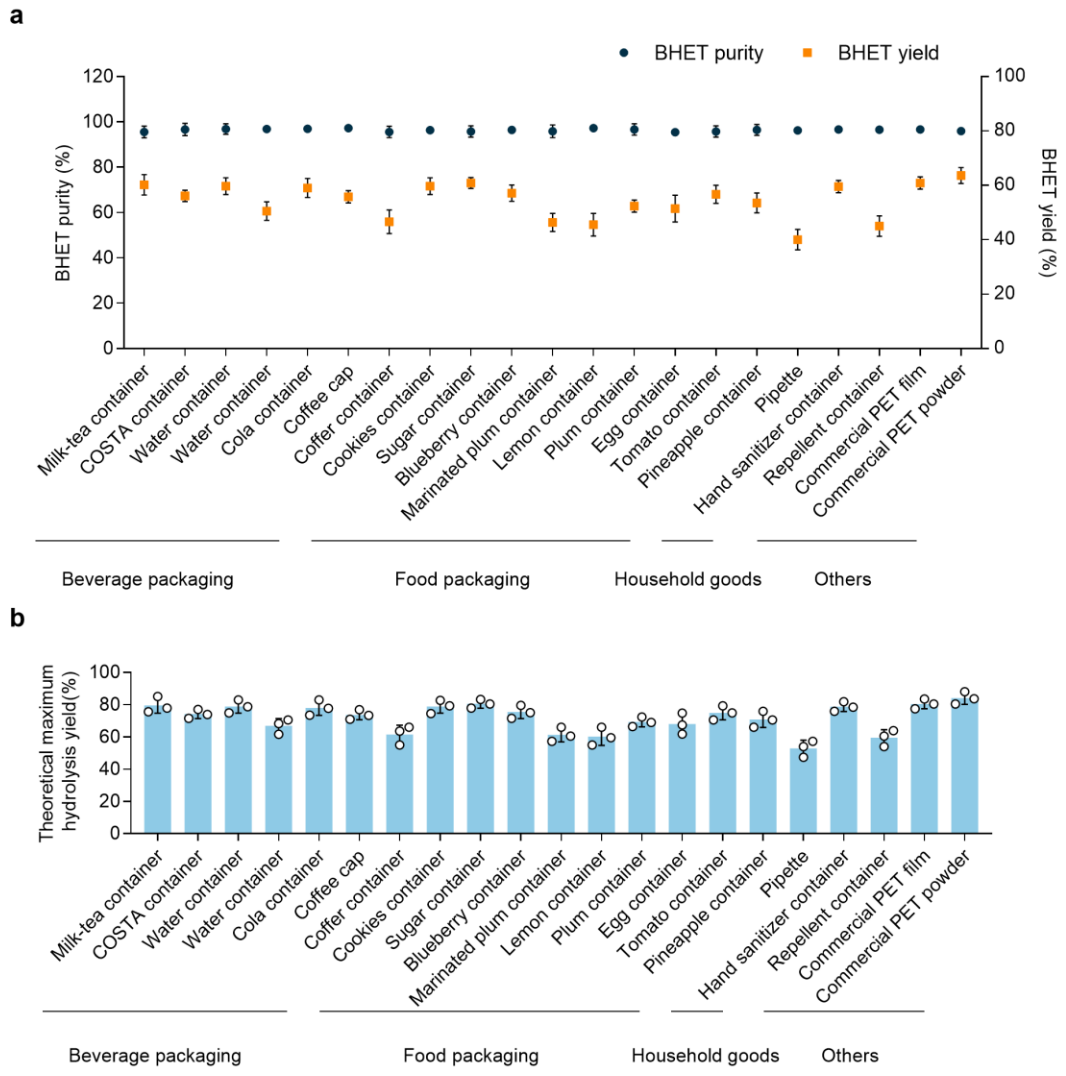

Supplementary Fig. 34 Depolymerization of commercial post-consumed PET products into BHET and upgrading of the pure monomers into a high-value derivative *p*-Phthaloyl chloride. (a) The BHET purity and BHET yield of 21 commercial post-consumed plastic products were obtained through chemical glycolysis. Reaction condition: the chemical glycolysis was performed at 210 °C for 3 h using K_2CO_3 as catalyst, and the pure BHET was obtained through filtration and recrystallization. (b) Theoretical maximum hydrolysis yield of TPA through a simple one-step chemical method. Reaction condition: 1.5 g of terephthalic acid, 4.4 g of thionyl chloride and 0.128 g of benzyl triethylammonium chloride (TEBAC) heated to reflux at 85 °C for 4

h. Error bars correspond to the standard deviation (s.d.) of three measurements (n=3). Source data are provided as a Source Data file.

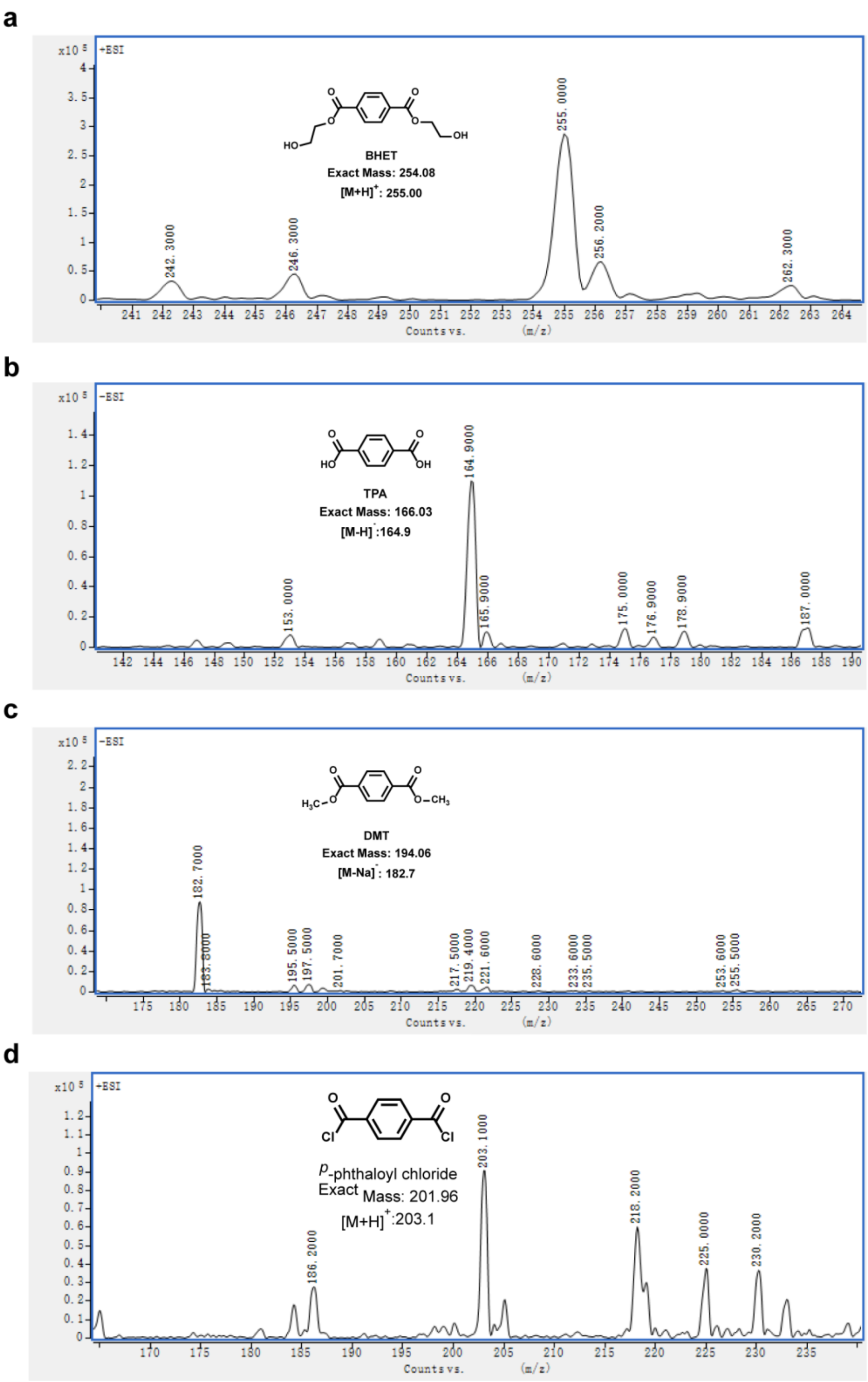

Supplementary Fig. 35 Liquid chromatography-mass spectrometry (LC-MS) data of BHET, TPA, and DMT. (a) LC-MS data of BHET by chemical glycolysis; (b) LC-MS data of TPA after enzymatic catalysis of truncated variants; (c) LC-MS data of DMT synthesized from TPA. (d) LC-MS data of *p*-phthaloyl chloride synthesized from TPA.

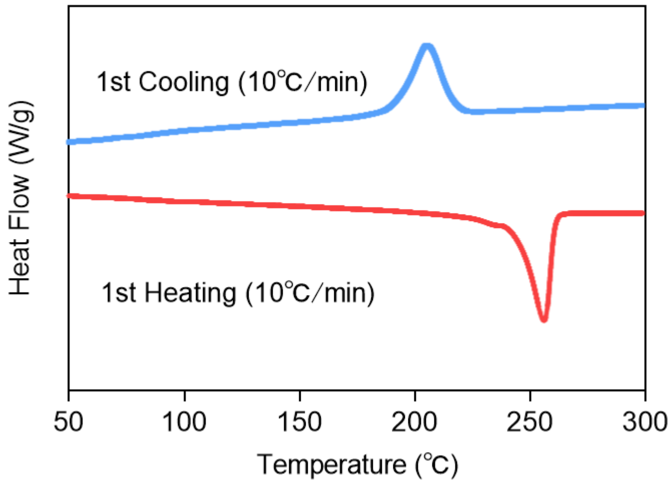

Supplementary Fig. 36 DSC trace of virgin PET regenerated from the degraded solutions. The crystallinity of this regenerated PET is 45.78%. The melting onset is 210.49 °C. The melting peak temperature is 255.99 °C. Source data are provided as a Source Data file.

Supplementary Table 1. Summary and classification of strains with over 55 μg/L MHET.

Genus	Strain NO.	Strains	Source	Plastic types
Bacillus	37, 70	*Bacillus subtilis*	Landfill	PET
Bacillus	39	*Bacillus aryabhattai*	Landfill	PET
Bacillus	18	*Bacillus marisflavi*	Landfill	PET
Bacillus	23	*Bacillus megaterium*	Landfill	PET
Klebsiella	36, 51	*Klebsiella sp.*	Landfill	PET
Chryseobacterium	29	*Chryseobacterium sp.*	Landfill	PET
Pseudomonas	22	*Pseudomonas putida*	Landfill	PET
Enterobacter	46	*Enterobacter sp.*	Landfill	PET

Supplementary Table 2. The genome used to perform SSNs analysis in this study.

Strains	Homologous genome	Accession
Bacillus subtilis PET-86	*Bacillus subtilis* subsp. subtilis str. 168	CP053102
Chryseobacterium sp. PET-29	-	CP107053

Supplementary Table 3. Genome characteristics of strain *Chryseobacterium sp.* PET-29.

Characteristics	Value
Raw reads size (bp) in Illumina platform	6,639,316
Clean reads size (bp)	6,607,788
Sequencing depth	150
Total sequence length (bp) in Nanopore platform	1,000,002,109
GC content (%)	37.69
Chromosome size (bp)	
CDS in chromosome	3689
Total size (bp)	4,125,210
5S rRNAs	5
16S rRNAs	5
23S rRNAs	5
tRNAs	63
tmRNA	1
Protein coding genes	3645
Uniprot annotation (genome)	1664
COGs annotation (genome)	1246
GO annotation (genome)	1608
KEGG annotation (genome)	808
Tigrfam annotation (genome)	1868
Pfam annotation (genome)	2972
All annotated genes	3794

Supplementary Table 4. The enzyme in *Chryseobacterium sp.* PET-29 that are speculated to be involved in PET hydrolysis.

Suggested enzyme name	Protein_ID
Non-hemolytic phospholipase C	assembly_03248
Lipase 2	assembly_03765
Pectinesterase A	assembly_03587
Trifunctional nucleotide phosphoesterase protein YfkN	assembly_03648
Carboxylesterase NlhH	assembly_03768
Rhamnogalacturonan acetylesterase RhgT	assembly_03583
Carboxylesterase NlhH	assembly_03581
Pectinesterase A	assembly_03580
hamnogalacturonan acetylesterase Rhg	assembly_03579
Endo-1,4-beta-xylanase/feruloyl esterase	assembly_03518
Acyl carrier protein phosphodiesterase	assembly_03472
Para-nitrobenzyl esterase	assembly_03435
RNA 2',3'-cyclic phosphodiesterase	assembly_03267
Putative esterase	assembly_03211
3',5'-cyclic adenosine monophosphate phosphodiesterase CpdA	assembly_03028
Acyl-coenzyme A thioesterase PaaI	assembly_02385
Ferri-bacillibactin esterase BesA	assembly_02381
3',5'-cyclic adenosine monophosphate phosphodiesterase CpdA	assembly_02268
Long-chain acyl-CoA thioesterase FadM	assembly_02188
Cocaine esterase	assembly_02039
Putative esterase	assembly_01895
Esterase YbfF	assembly_01871
Phosphoribosyl 1,2-cyclic phosphate phosphodiesterase	assembly_01778
3',5'-cyclic adenosine monophosphate phosphodiesterase CpdA	assembly_01562
Putative esterase	assembly_01533
Rhamnogalacturonan acetylesterase RhgT	assembly_01248
Acetylxylan esterase	assembly_01230
Carbohydrate acetyl esterase/feruloyl esterase	assembly_01137
Arylesterase	assembly_01099
Acetylxylan esterase	assembly_01081
Carbohydrate acetyl esterase/feruloyl esterase	assembly_01080
Chemotaxis response regulator protein-glutamate methylesterase	assembly_00986
Carbohydrate acetyl esterase/feruloyl esterase	assembly_00655
3',5'-cyclic adenosine monophosphate phosphodiesterase CpdA	assembly_00188
Carboxylesterase 2	assembly_00100
3',5'-cyclic adenosine monophosphate phosphodiesterase CpdA	assembly_00043
Glycerophosphodiester phosphodiesterase	ssembly_00040

Supplementary Table 5. The enzymes in *Bacillus subtilis* PET-86 that are speculated to be involved in PET hydrolysis.

Enzyme name	Protein_ID
lipase LipA	QJR44727.1
spore germination lipase LipC	QJR44868.1
patatin-like phospholipase family protein	QJR47032.1
phospholipase YtpA	QJR47633.1
glycerophosphodiester phosphodiesterase	QJR44671.1
enantioselective carboxylesterase CesB	QJR44682.1
acetylxylan esterase	QJR44774.1
surfactin biosynthesis thioesterase SrfAD	QJR44807.1
carboxylesterase	QJR45001.1
rhamnogalacturonan acetylesterase	QJR45161.1
rhamnogalacturonan acetylesterase	QJR45166.1
esterase EstB	QJR45288.1
glycerophosphodiester phosphodiesterase	QJR45406.1
esterase family protein	QJR45625.1
acyl-CoA thioesterase	QJR48713.1
metallophosphoesterase	QJR45784.1
metallophosphoesterase	QJR45855.1
2',3'-cyclic-nucleotide 2'-phosphodiesterase	QJR46144.1
YbgC/FadM family acyl-CoA thioesterase	QJR46252.1
metallophosphoesterase	QJR46881.1
glycerophosphodiester phosphodiesterase	QJR46998.1
cyclic-di-AMP phosphodiesterase PgpH	QJR47110.1
metallophosphoesterase	QJR47423.1
acyl-CoA thioesterase	QJR47430.1
RNA 2',3'-cyclic phosphodiesterase	QJR47582.1
hotdog fold thioesterase	QJR47752.1
cyclic di-GMP phosphodiesterase	QJR47760.1
ferri-bacillibactin esterase BesA	QJR47784.1
carboxylesterase	QJR47945.1
para-nitrobenzyl esterase	QJR48005.1
esterase	QJR48472.1
metallophosphoesterase	QJR48582.1
cyclic-di-AMP phosphodiesterase GdpP	QJR48614.1
alpha/beta hydrolase	QJR44576.1
alpha/beta hydrolase	QJR44620.1
alpha/beta hydrolase	QJR44657.1
alpha/beta hydrolase	QJR44780.1
alpha/beta hydrolase	QJR44821.1
alpha/beta hydrolase	QJR45086.1
alpha/beta hydrolase	QJR45310.1
alpha/beta hydrolase	QJR45529.1
alpha/beta hydrolase	QJR46411.1
alpha/beta hydrolase	QJR46946.1
alpha/beta hydrolase	QJR46965.1
alpha/beta hydrolase	QJR47274.1
alpha/beta hydrolase	QJR47274.1
alpha/beta hydrolase	QJR47726.1
alpha/beta hydrolase	QJR47947.1

Supplementary Table 6. Common structural features of PET hydrolases.

Characterstics	Lipase	Esterase	Cutinase	PETase
Superfamily	α/β hydrolase	α/β hydrolase	α/β hydrolase	α/β hydrolase
Lid-domain	+	+	-	-
Catalytic triad (Ser-Asp/Glu-His)	+	+	+	+
Disulfide bonds/bridges	-	-	+	+
Substrate-binding cleft	Narrow/deep	Narrow/deep	Wide/shallow	Wide/shallow
PDB ID	1TCA, 2DSN, 1HQD, 3RLI, 1JI3, 1EX,9 2ZYH,1JFR,	5AH1, 5A2G, 6AID, 5D8M, 1Q3E,	4OYY, 3VIS, 4WFJ, 1CUS, 5LUJ, 3DCN, 3GBS, 4CG1, 4EB0, 1CEX,	5XG0

Supplementary Table 7. Twenty-nine reported PET hydrolases are used to sequence similarity networks.

No.	Reported PET hydrolases	Ref
1	PETase(5XGO)	[7]
2	SvCut (4WFI)	[20]
3	BTA-hydrolase 1	[21]
4	BTA-hydrolase 2	[21]
5	Thh_Est	[22]
6	Thf42_Cut1	[23]
7	SeL (1JFR)	[24]
8	Est119 (3VIS)	[25]
9	Tcur0390	[26]
10	PaPL (6SBN)	[27]
11	Ta_cut (6AID)	[28]
12	Tcur1278	[29]
13	Cut190(7CTS)	[30]
14	Thc_Cut2 (5LUL)	[31]
15	Cutinase (4CG1)	[32]
16	Tha_Cut1	[33]
17	LCC (4EB0)	[34]
18	Cutinase (4OYY)	[35]
19	Cutinase (1CUS)	[36]
20	Cutinase (3DCN)	[37]
21	Cutinase (3GBS)	[38]
22	Cutinase (1CEX)	[39]
23	Bs2Est (1QE3)	[6]
24	Chath_Est1(5A2G)	[5]
25	Lipase (2DSN)	[40]
26	Cbotu_EstA(5AH1)	[41]
27	Lipase (1JI3)	[42]
29	Lipase (1HQD)	[43]
29	Lipase (1EX9)	[44]

Supplementary Table 8. Information of enzymes cloned in this study

Enzyme	Strain	Abbreviation	AA[a]	Catalytic Triad Residues
para-nitrobenzyl esterase	*Bacillus subtilis* PET-86	BsEst	489	S189-E310-H399
para-nitrobenzyl esterase	*Chryseobacterium sp.* PET-29	ChryBHETase	445	S194-E310-H379

[a] Sequence information for all enzymes is available in Supplementary Information.

Supplementary Table 9. Half-life of BHETases and ΔBHETases at different temperatures[a].

	50 °C	55 °C	60 °C	65 °C	70 °C
BsEst	89.64±4.12 h	62.36±6.63 h	33.07±2.57 h	7.88±0.67 h	0.65±0.04 h
ΔBsEst	90.07±4.99 h	60.36±2.04 h	38.23±2.19 h	9.57±0.74 h	0.97±0.13 h
ChryBHETase	1.81±0.15 h	0.40±0.04 h	0.17±0.03h	0.10±0.02 h	0.04±0.01 h
ΔChryBHETase	1.92±0.47 h	0.66±0.04 h	0.18±0.03 h	0.09±0.01 h	0.05±0.01 h

[a] All reactions were carried out in 50 mM potassium phosphate buffer (pH 7.5) at 30 °C using BHET as substrate. Among them, the purified proteins were stored separately at 50-70°C (at 5°C intervals) before detecting enzyme activity. The values were averaged from three replicates (n=3) with the standard deviation (s.d.). Source data are provided as a Source Data file.

Supplementary Table 10. Summary of calculated observables during MD simulation.

Descriptor	Location	Observables	Results
Geometrical property	Overall protein	Time-averaged RMSD	S13a, S13b
		Time-averaged Rg	S14a, S14b
		Internal H-bond	S15a, S15b
		Total SASA<br>Hydrophobic SASA<br>Hydrophilic SASA	S16a, S16b,<br>S17a, S17b
		RMSF of residue	Fig 2h, S25
	Barrier region	B-factor	S21
	SBC	Electrostatic potential energy distribution	Fig 2g, S30
		RMSF	S29
	Active site	Distance of Ser189-HG with His399-NE2<br>Distance of Glu310-OE2 with His399-HD1	S19a, S19b
		Distance of Ser194-HG with His379-NE2<br>Distance of Glu310-OE2 with His379-HD1	S20a, S20b
Solvation phenomenon	Overall protein	Spatial distribution of BHET and water	Fig 3b
	Barrier region	Number of BHET/water molecule	S18
	Active site	Number of BHET/water molecule	Fig 2i, 4g, and 4h
		Distance of BHET with active site	Fig 4c-f and S26
In total		20	

Supplementary Table 11. Twenty-one commercial post-consumed plastic products collected in this study [a].

Category	Sample number	Post-consumer plastic products	Initial mass (g)	Crystallinity %
Beverage packaging	#1	Milk-tea container	5.00	6.39
	#2	COSTA container	5.00	12.30
	#3	Water container	5.00	2.70
	#4	Water container	5.00	4.40
	#5	Cola container	5.00	1.72
	#6	Coffee cap	5.00	2.89
	#7	Coffer container	5.00	10.76
Food packaging	#8	Cookies container	5.00	5.37
	#9	Sugar container	5.00	5.59
	#10	Blueberry container	5.00	38.73
	#11	Marinated plum container	5.00	0.07
	#12	Lemon container	5.00	5.39
	#13	Plum container	5.00	15.15
	#14	Egg container	5.00	5.42
	#15	Tomato container	5.00	7.23
	#16	Pineapple container	5.00	5.03
	#17	Pipette	5.00	3.31
Household goods packaging	#18	Hand sanitizer container	5.00	13.71
	#19	Repellent container	5.00	2.51
Others	#20	Commercial PET film	5.00	4.46
	#21	Commercial PET powder	5.00	1.54

[a]21 different post-consumed plastic products used in the packaging of food, beverages, medications, office supplies, household goods, and cosmetics available at local grocery store chains. The crystallinity % of the intact pc-PET films was determined by DSC. All measurements were conducted in triplicate (n = 3), and the mean values were presented.

Supplementary Table 12. The primers of target gene cloning and pET-22b(+) vector.

Primer	Sequence (5'-3')
BS-F	GCGATGGCCATGGATATCATGACTCATCAAATAG
BS-R	CTCGAGTGCGGCTTCTCCTTTTGAAGGGAATA
BS-pET-22b-F	AAGCCGCACTCGAGCACCACCACCACCACCACTGAGAT
BS-pET-22b-R	CATGATATCCATGGCCATCGCCGGCTGGGCAGCGA
Chry sp.-F	TGGATATCATGACAACACAGCAACACAAGAAAATC
Chry sp.-R	TGCTCGAGTGCGGCATTCTTAACTTTTTTAAGC
Chry sp.-pET-22b-F	AATGCCGCACTCGAGCACCACCACCACCACCACTGAGA
Chry sp.-pET-22b-R	TGTTGTCATGATATCCATGGCCATCGCCGGC

Supplementary References

1. Maheswaran, B. *et al.* In vivo degradation of polyethylene terephthalate using microbial isolates from plastic polluted environment. *Chemosphere* **310**, 136757 (2023).
2. Gricajeva, A., Nadda, A. K. & Gudiukaite, R. Insights into polyester plastic biodegradation by carboxyl ester hydrolases. *J. Chem. Technol. Biotechnol.* **97**, 359–380 (2022).
3. Zallot, R., Oberg, N. & Gerlt, J. A. Discovery of new enzymatic functions and metabolic pathways using genomic enzymology web tools. *Curr. Opin. Biotechnol.* **69**, 77–90 (2021).
4. Kim, H. T. *et al.* Chemo-Biological Upcycling of Poly(ethylene terephthalate) to Multifunctional Coating Materials. *ChemSusChem.* **14**, 4251–4259 (2021).
5. Perz, V. *et al.* An Esterase from Anaerobic Clostridium hathewayi Can Hydrolyze Aliphatic–Aromatic Polyesters. *Environ. Sci. Technol.* **50**, 2899–2907 (2016).
6. Zock, J. *et al.* The Bacillus subtilis pnbA gene encoding p-nitrobenzyl esterase: cloning, sequence and high-level expression in Escherichia coli. *Gene* **151**, 37–43 (1994).
7. Yoshida, S. *et al.* A bacterium that degrades and assimilates poly(ethylene terephthalate). *Science* **351**, 1196–1199 (2016).
8. Pinto, A. V. *et al.* Reaction Mechanism of MHETase, a PET Degrading Enzyme. *ACS Catal.* **11**, 10416–10428 (2021).
9. Bollinger, A. *et al.* A Novel Polyester Hydrolase From the Marine Bacterium Pseudomonas aestusnigri – Structural and Functional Insights. *Front. Microbiol.* **11**, (2020).
10. Araújo, R. *et al.* Tailoring cutinase activity towards polyethylene terephthalate and polyamide 6,6 fibers. *J. Biotechnol.* **128**, 849–857 (2007).
11. Mathesh, M. *et al.* Opening Lids: Modulation of Lipase Immobilization by Graphene Oxides. *ACS Catal.* **6**, 4760–4768 (2016).
12. Tang, Q., Lan, D., Yang, B., Khan, F. I. & Wang, Y. Site-directed mutagenesis studies of hydrophobic residues in the lid region of T1 lipase. *Eur. J. Lipid Sci. Technol.* **119**, 1600107 (2017).
13. Bell, E. L. *et al.* Directed evolution of an efficient and thermostable PET depolymerase. *Nat. Catal.* **5**, 673–681 (2022).
14. Tournier, V. *et al.* An engineered PET depolymerase to break down and recycle plastic bottles. *Nature* **580**, 216–219 (2020).
15. Lu, H. *et al.* Machine learning-aided engineering of hydrolases for PET depolymerization. *Nature* **604**, 662–667 (2022).
16. Cui, Y. *et al.* Computational redesign of a PETase for plastic biodegradation under ambient condition by the GRAPE strategy. *ACS Catal.* **11**, 1340–1350 (2021).
17. Shi, L. *et al.* Complete depolymerization of PET waste by an evolved PET hydrolase from directed evolution. *Angew. Chem. Int. Ed.* **62**, 1–11 (2023).
18. Rose, N. C. Preparation of terephthaloyl chloride: Prelude to ersatz Nylon. *J. Chem. Educ.* **44**, 283 (1967).

19. Meys, R. *et al.* Achieving net-zero greenhouse gas emission plastics by a circular carbon economy. *Science* **374**, 71–76 (2021).
20. Miyakawa, T. *et al.* Structural basis for the Ca(2+)-enhanced thermostability and activity of PET-degrading cutinase-like enzyme from Saccharomonospora viridis AHK190. *Appl. Microbiol. Biotechnol.* **99**, 4297–4307 (2015).
21. Lameh, F., Baseer, A. Q. & Ashiru, A. G. Comparative molecular docking and molecular-dynamic simulation of wild-type- and mutant carboxylesterase with BTA-hydrolase for enhanced binding to plastic. *Eng. Life Sci.* **22**, 13–29 (2022).
22. Ribitsch, D. *et al.* A new esterase from *Thermobifida halotolerans* hydrolyses polyethylene terephthalate (PET) and polylactic acid (PLA). *Polymers* **4**, 617–629 (2012).
23. Herrero Acero, E. *et al.* Enzymatic surface hydrolysis of PET: Effect of structural diversity on kinetic properties of cutinases from *Thermobifida*. *Macromol.* **44**, 4632–4640 (2011).
24. Wei, Y. *et al.* Structure of a microbial homologue of mammalian platelet-activating factor acetylhydrolases: Streptomyces exfoliatus lipase at 1.9 A resolution. *Struct.* **6**, 511–519 (1998).
25. Ohta, T., Horie, H., Matsu-ura, A. & Kawai, F. Cloning, expression, and characterization of novel GH5 endoglucanases from Thermobifida alba AHK119. *J. Biosci. Bioeng.* **127**, 554–562 (2019).
26. Wei, R. *et al.* Functional characterization and structural modeling of synthetic polyester-degrading hydrolases from *Thermomonospora curvata*. *AMB. Expr.* **4**, 44 (2014).
27. Bollinger, A. *et al.* A Novel Polyester Hydrolase From the Marine Bacterium Pseudomonas aestusnigri - Structural and Functional Insights. *Front. Microbiol.* **11**, 114 (2020).
28. Kitadokoro, K. *et al.* Structural insights into the unique polylactate-degrading mechanism of Thermobifida alba cutinase. *FEBS J.* **286**, 2087–2098 (2019).
29. Islam, S., Apitius, L., Jakob, F. & Schwaneberg, U. Targeting microplastic particles in the void of diluted suspensions. *Environ. Int.* **123**, 428–435 (2019).
30. Emori, M. *et al.* Structural basis of mutants of PET-degrading enzyme from Saccharomonospora viridis AHK190 with high activity and thermal stability. *Proteins* **89**, 502–511 (2021).
31. Ribitsch, D. *et al.* Small cause, large effect: Structural characterization of cutinases from Thermobifida cellulosilytica. *Biotechnol. Bioeng.* **114**, 2481–2488 (2017).
32. Roth, C. *et al.* Structural and functional studies on a thermostable polyethylene terephthalate degrading hydrolase from Thermobifida fusca. *Appl. Microbiol. Biotechnol.* **98**, 7815–7823 (2014).
33. Ribitsch, D. *et al.* Characterization of a new cutinase from *Thermobifida alba* for PET-surface hydrolysis. *Biocatal. Biotransform.* **30**, 2–9 (2012).

34. Sulaiman, S. *et al.* Isolation of a novel cutinase homolog with polyethylene terephthalate-degrading activity from leaf-branch compost by using a metagenomic approach. *Appl. Environ. Microbiol.* **78**, 1556–1562 (2012).
35. Kold, D. *et al.* Thermodynamic and structural investigation of the specific SDS binding of Humicola insolens cutinase. *Protein Sci.* **23**, 1023–1035 (2014).
36. Martinez, C., De Geus, P., Lauwereys, M., Matthyssens, G. & Cambillau, C. Fusarium solani cutinase is a lipolytic enzyme with a catalytic serine accessible to solvent. *Nature* **356**, 615–618 (1992).
37. Nyon, M. P. *et al.* Catalysis by Glomerella cingulata cutinase requires conformational cycling between the active and inactive states of its catalytic triad. *J. Mol. Biol.* **385**, 226–235 (2009).
38. Liu, Z. *et al.* Structural and functional studies of Aspergillus oryzae cutinase: enhanced thermostability and hydrolytic activity of synthetic ester and polyester degradation. *J. Am. Chem. Soc.* **131**, 15711–15716 (2009).
39. Longhi, S., Czjzek, M., Lamzin, V., Nicolas, A. & Cambillau, C. Atomic resolution (1.0 A) crystal structure of Fusarium solani cutinase: stereochemical analysis. *J. Mol. Biol.* **268**, 779–799 (1997).
40. Matsumura, H. *et al.* Novel cation-pi interaction revealed by crystal structure of thermoalkalophilic lipase. *Proteins* **70**, 592–598 (2008).
41. Perz, V. *et al.* Hydrolysis of synthetic polyesters by Clostridium botulinum esterases. *Biotechnol. Bioeng.* **113**, 1024–1034 (2016).
42. Tyndall, J. D. A., Sinchaikul, S., Fothergill-Gilmore, L. A., Taylor, P. & Walkinshaw, M. D. Crystal structure of a thermostable lipase from Bacillus stearothermophilus P1. *J. Mol. Biol.* **323**, 859–869 (2002).
43. Luić, M. *et al.* Complex of Burkholderia cepacia lipase with transition state analogue of 1-phenoxy-2-acetoxybutane: biocatalytic, structural and modelling study. *Eur. J. Biochem.* **268**, 3964–3973 (2001).
44. Nardini, M., Lang, D. A., Liebeton, K., Jaeger, K. E. & Dijkstra, B. W. Crystal structure of pseudomonas aeruginosa lipase in the open conformation. The prototype for family I.1 of bacterial lipases. *J. Biol. Chem.* **275**, 31219–31225 (2000).
