# Supplementary material for: Discovery and mechanism-guided engineering of BHET hydrolases for improved PET recycling and upcycling
Source: Nat Commun. 2023 Jul 13;14:4169. doi: 10.1038/s41467-023-39929-w (PMC10344914; doi:10.1038/s41467-023-39929-w)
Supplement: Supplementary file 3 — Reporting Summary [file 41467_2023_39929_MOESM3_ESM.pdf]

nature portfolio

Corresponding author(s): Xiujuan Li, He Huang

Last updated by author(s): Jun 21, 2023

# Reporting Summary

Nature Portfolio wishes to improve the reproducibility of the work that we publish. This form provides structure for consistency and transparency in reporting. For further information on Nature Portfolio policies, see our Editorial Policies and the Editorial Policy Checklist.

## Statistics

For all statistical analyses, confirm that the following items are present in the figure legend, table legend, main text, or Methods section.

| n/a | Confirmed | |
|---|---|---|
| ☐ | ☒ | The exact sample size (*n*) for each experimental group/condition, given as a discrete number and unit of measurement |
| ☐ | ☒ | A statement on whether measurements were taken from distinct samples or whether the same sample was measured repeatedly |
| ☒ | ☐ | The statistical test(s) used AND whether they are one- or two-sided<br>*Only common tests should be described solely by name; describe more complex techniques in the Methods section.* |
| ☒ | ☐ | A description of all covariates tested |
| ☒ | ☐ | A description of any assumptions or corrections, such as tests of normality and adjustment for multiple comparisons |
| ☐ | ☒ | A full description of the statistical parameters including central tendency (e.g. means) or other basic estimates (e.g. regression coefficient) AND variation (e.g. standard deviation) or associated estimates of uncertainty (e.g. confidence intervals) |
| ☒ | ☐ | For null hypothesis testing, the test statistic (e.g. *F*, *t*, *r*) with confidence intervals, effect sizes, degrees of freedom and *P* value noted<br>*Give P values as exact values whenever suitable.* |
| ☒ | ☐ | For Bayesian analysis, information on the choice of priors and Markov chain Monte Carlo settings |
| ☒ | ☐ | For hierarchical and complex designs, identification of the appropriate level for tests and full reporting of outcomes |
| ☒ | ☐ | Estimates of effect sizes (e.g. Cohen's *d*, Pearson's *r*), indicating how they were calculated |

*Our web collection on statistics for biologists contains articles on many of the points above.*

## Software and code

Policy information about availability of computer code

| | |
|---|---|
| Data collection | Chromoleon software was used for acquisition of spectra on Agilent 1200 and Ultimate 3000 UHPLC systems.<br>The crystallinity of the PET films was determined by NETZSCH DSC 214.<br>The water contact angle was determined by Contact Angle Meter OCA20<br>The SEM images was obtained by Scanning Electron Microscope (SEM S4800).<br>Sequence similarity networks (SSNs) analysis was performed by EFI-EST (EFI DATABASE VERSION: 2023_02 / 94)<br>The KEGG (VERSION: 87.0-r20180701), COG (VERSION: 2014), and GO (VERSION: 2014) databases were used for functional annotation. |
| Data analysis | The AlphaFold2 was used for predicting the protein structure.<br>Autodock Vina was used to generate enzyme-ligand complexes,and the docking results were then visualized and analyzed by Pymol 2.5.2.<br>Molecular Dynamics(MD)simulation and analysis were performed with GROMACS 2016 simulation package with the GROMOS96(54a7)force ield.<br>Protein multiple alignment were done using Clustal Omega 1.2.2.<br>All experimental data were analyzed by Graphpad Prism 8 version 8.4.3.<br>The assembled genome was predicted by Prokka 1.12 software. |

For manuscripts utilizing custom algorithms or software that are central to the research but not yet described in published literature, software must be made available to editors and reviewers. We strongly encourage code deposition in a community repository (e.g. GitHub). See the Nature Portfolio guidelines for submitting code & software for further information.

## Data

Policy information about availability of data

All manuscripts must include a data availability statement. This statement should provide the following information, where applicable:

- Accession codes, unique identifiers, or web links for publicly available datasets
- A description of any restrictions on data availability
- For clinical datasets or third party data, please ensure that the statement adheres to our policy

The 16S rRNA of reported strains have been deposited in the GenBank under accession codes as follows: Bacillus subtilis PET-86, OP564167; Chryseobacterium sp. PET-29, OP564169; genome information of Chryseobacterium sp. PET-29, CP107053 (ASM2573681v1 - Genome - Assembly - NCBI (nih.gov)). The nucleotides of reported enzymes have been deposited in the GenBank under accession codes as follows: BsEst, OR147951; ChryBHETase, OR147952.

## Research involving human participants, their data, or biological material

Policy information about studies with human participants or human data. See also policy information about sex, gender (identity/presentation), and sexual orientation and race, ethnicity and racism.

| | |
|---|---|
| Reporting on sex and gender | N/A |
| Reporting on race, ethnicity, or other socially relevant groupings | N/A |
| Population characteristics | N/A |
| Recruitment | N/A |
| Ethics oversight | N/A |

Note that full information on the approval of the study protocol must also be provided in the manuscript.

# Field-specific reporting

Please select the one below that is the best fit for your research. If you are not sure, read the appropriate sections before making your selection.

☒ Life sciences ☐ Behavioural & social sciences ☐ Ecological, evolutionary & environmental sciences

For a reference copy of the document with all sections, see nature.com/documents/nr-reporting-summary-flat.pdf

# Life sciences study design

All studies must disclose on these points even when the disclosure is negative.

| | |
|---|---|
| Sample size | The sample size follows common standards employing three or more biological replicates. All sample sizes are listed in each figure legend. |
| Data exclusions | No data was excluded. |
| Replication | All in vitro and In silico experiments with explicit standard deviation (s.d.) were performed in triplicates. Attempts at replication were successful. |
| Randomization | Samples were randomly allocated in this study. |
| Blinding | The investigators were blinded to group allocation during data collection and analysis. |

# Reporting for specific materials, systems and methods

We require information from authors about some types of materials, experimental systems and methods used in many studies. Here, indicate whether each material, system or method listed is relevant to your study. If you are not sure if a list item applies to your research, read the appropriate section before selecting a response.

## Materials & experimental systems

| n/a | Involved in the study |
| --- | --- |
| ☒ | ☐ Antibodies |
| ☒ | ☐ Eukaryotic cell lines |
| ☒ | ☐ Palaeontology and archaeology |
| ☒ | ☐ Animals and other organisms |
| ☒ | ☐ Clinical data |
| ☒ | ☐ Dual use research of concern |
| ☒ | ☐ Plants |

## Methods

| n/a | Involved in the study |
| --- | --- |
| ☒ | ☐ ChIP-seq |
| ☒ | ☐ Flow cytometry |
| ☒ | ☐ MRI-based neuroimaging |
